# Acquisition of C1 inhibitor by *Bordetella pertussis* virulence associated gene 8 results in C2 and C4 consumption away from the bacterial surface

**DOI:** 10.1371/journal.ppat.1006531

**Published:** 2017-07-24

**Authors:** Elise S. Hovingh, Bryan van den Broek, Betsy Kuipers, Elena Pinelli, Suzan H. M. Rooijakkers, Ilse Jongerius

**Affiliations:** 1 Department of Medical Microbiology, University Medical Center Utrecht, Utrecht, The Netherlands; 2 Centre for Infectious Disease Control, National institute for Public Health and the Environment, Bilthoven, The Netherlands; University of California Davis School of Medicine, UNITED STATES

## Abstract

Whooping cough, or pertussis, is a contagious disease of the respiratory tract that is re-emerging worldwide despite high vaccination coverage. The causative agent of this disease is the Gram-negative *Bordetella pertussis*. Knowledge on complement evasion strategies of this pathogen is limited. However, this is of great importance for future vaccine development as it has become apparent that a novel pertussis vaccine is needed. Here, we unravel the effect of Virulence associated gene 8 (Vag8) of *B*. *pertussis* on the human complement system at the molecular level. We show that both recombinant and endogenously secreted Vag8 inhibit complement deposition on the bacterial surface at the level of C4b. We reveal that Vag8 binding to human C1-inhibitor (C1-inh) interferes with the binding of C1-inh to C1s, C1r and MASP-2, resulting in the release of active proteases that subsequently cleave C2 and C4 away from the bacterial surface. We demonstrate that the depletion of these complement components in the bacterial surrounding and subsequent decreased deposition on *B*. *pertussis* leads to less complement-mediated bacterial killing. Vag8 is the first protein described that specifically prevents C1s, C1r and MASP-2 binding to C1-inh and thereby mediates complement consumption away from the bacterial surface. Unravelling the mechanism of this unique complement evasion strategy of *B*. *pertussis* is one of the first steps towards understanding the interactions between the first line of defense complement and *B*. *pertussis*.

## Author summary

Despite wide-spread vaccination, whooping cough caused by the Gram-negative bacterium *Bordetella pertussis* remains a public health problem and has been re-emerging in the past decades. To this end, new vaccination strategies are being explored including the use of complement evasion molecules as vaccine candidates. Autotransporter Virulence associated gene 8 (Vag8) was previously shown to be involved in complement evasion. However, the molecular mechanism of this immune evasion was not understood. Considering knowledge on molecular mechanisms is crucial for further studies regarding vaccine development, we investigated the underlying mechanism of Vag8 induced complement evasion of *B*. *pertussis*. We show that both recombinant Vag8 as well as endogenously secreted Vag8 inhibits complement activation via the classical and lectin complement pathway at the level of C4 and C2. We identified a novel bacterial complement evasion strategy initiated by the binding of Vag8 to C1-inhibitor. This binding interferes with the interactions between C1-inhibitor and the proteases C1s, C1r and MASP-2 resulting in the release of active proteases that cleave C4 and C2 away from the bacterial surface. This environmental consumption of C4 and C2 leads to decreased complement deposition on the bacterial surface and hence inhibits complement-mediated killing of *B*. *pertussis*.

## Introduction

The Gram-negative *Bordetella pertussis* is the causative agent of whooping cough, or pertussis, which despite high vaccine coverage has been re-emerging in the past decades posing a continuous global health problem. One of the possible reasons for this re-emergence is pathogen adaptation [1]. At present, over 90% of the circulating strains carry the pertussis toxin promotor (*ptxP)* 3 allele which has replaced the previously circulating *ptxP*2 strains on which our current vaccines are based [1, 2]. These *ptxP3* strains express more pertussis toxin (Ptx) and have been shown to be associated with increased severity of disease [3, 4]. Virulence associated gene 8 (Vag8) is an autotransporter of *B*. *pertussis* that is synthesized as a 95 kD precursor protein and is further processed to a channel and a passenger domain [5]. The passenger domain of autotransporters can subsequently be cleaved and thus secreted by the bacteria. Alternatively, the passenger domain can be retained on the bacterial membrane as well as be present on outer membrane vesicles (OMVs). It was previously shown, that 34% of the protein content of OMVs is Vag8 [6]. The approximately 60 kDa passenger domain of Vag8 has been shown to bind C1-inhibitor (C1-inh) and has been suggested to be involved in complement evasion [7]. It was shown that a *vag8* mutant was more susceptible to complement-mediated killing compared to the isogenic wild type strain, however, the molecular mechanism of Vag8 mediated complement evasion of *B*. *pertussis* was not studied [7]. Interestingly, Vag8 is expressed 1.7 to 3.8 fold more by the newly emerging *ptxP3* strains compared to older strains [8, 9]. The need for a novel or improved pertussis vaccine has become evident due to re-emergence of this vaccine preventable disease in the past decades [10]. To this end, complement evasion molecules, such as Vag8, have been proposed as potential vaccine components [11, 12]. However, to evaluate the potential of Vag8 to serve as a vaccine candidate, it is important to understand its molecular mechanism.

The complement system, active mainly in plasma but also on respiratory mucosal surfaces, is the first line of defence against invading pathogens [13]. Complement can be activated via three different pathways: the classical pathway (CP), the lectin pathway (LP) and the alternative pathway (AP) [14]. The CP is initiated by the recognition of antigen-antibody complexes by the C1 complex [15, 16]. The proteases of the active C1 complex cleave C4 and C2 resulting in the formation of the C3 convertase (C4b2a) [17]. Activation of the LP is initiated by the recognition of sugar patterns by mannose-binding lectin (MBL), ficollins and collectins, which are associated with MBL-associated serine proteases (MASPs) [18]. The proteases of the MBL/MASP complex, which has an architecture similar to the C1 complex, can also cleave C4 and C2 giving rise to the C3 convertase (C4b2a) [18–21]. The AP can be activated spontaneously and additionally functions as an amplification loop for the former two pathways [14]. Upon its activation, the specific AP C3 convertase (C3bBb) is formed [14]. Cleavage of C3 by either one of the C3 convertases results in the generation of C3b, which deposits on the bacterial membrane and is important for bacterial phagocytosis as well as the formation of the C5 convertase of the CP/LP (C4b2aC3b) and the C5 convertase of the AP (C3bBbC3b) [22, 23]. The C5 convertases can cleave C5 resulting in the generation of the chemoattractant C5a and C5b which is the first building block of the membrane attack complex (MAC) [22]. The MAC forms a pore in the bacterial membrane of Gram-negative bacteria and subsequently results in bacterial killing [24].

Evasion of the complement system is a wide spread survival strategy used by many bacterial pathogens [25]. Bacteria are known to implement different mechanism to circumvent complement-mediated killing such as the secretion of small evasion molecules, as well as proteases that can cleave complement proteins. Bacteria can also stimulate regulatory cross-talk between Toll-like and complement receptors or recruit host complement fluid phase regulatory proteins to the bacterial surface [26–29]. One of these fluid phase regulatory proteins is the 105 kDa C1-inh, which is the major negative regulator of the CP and LP inactivating the proteases [30]. Various pathogens have been shown to express proteins that can bind C1-inh as a complement evasion strategy including Vag8 of *Bordetella pertussis* [28, 31]. Here, we characterized the underlying mechanism of complement evasion by *B*. *pertussis* Vag8. We show that Vag8 specifically targets the CP and the LP while the AP is unaffected. Both recombinant and endogenously secreted Vag8 inhibit complement deposition on the bacterial membrane, subsequently preventing bacterial complement-mediated killing. We identified a novel complement evasion strategy in which Vag8 interferes with the interaction of C1-inh with the C1 and MBL/MASP complex proteases, leading to the release of active proteases, which cleave C4 and C2 away from the bacterial surface.

## Results

### The Vag8 passenger domain is secreted during liquid growth, binds C1-inh and mediates resistance to complement-mediated killing of *B*. *pertussis*

**Fig 1:**
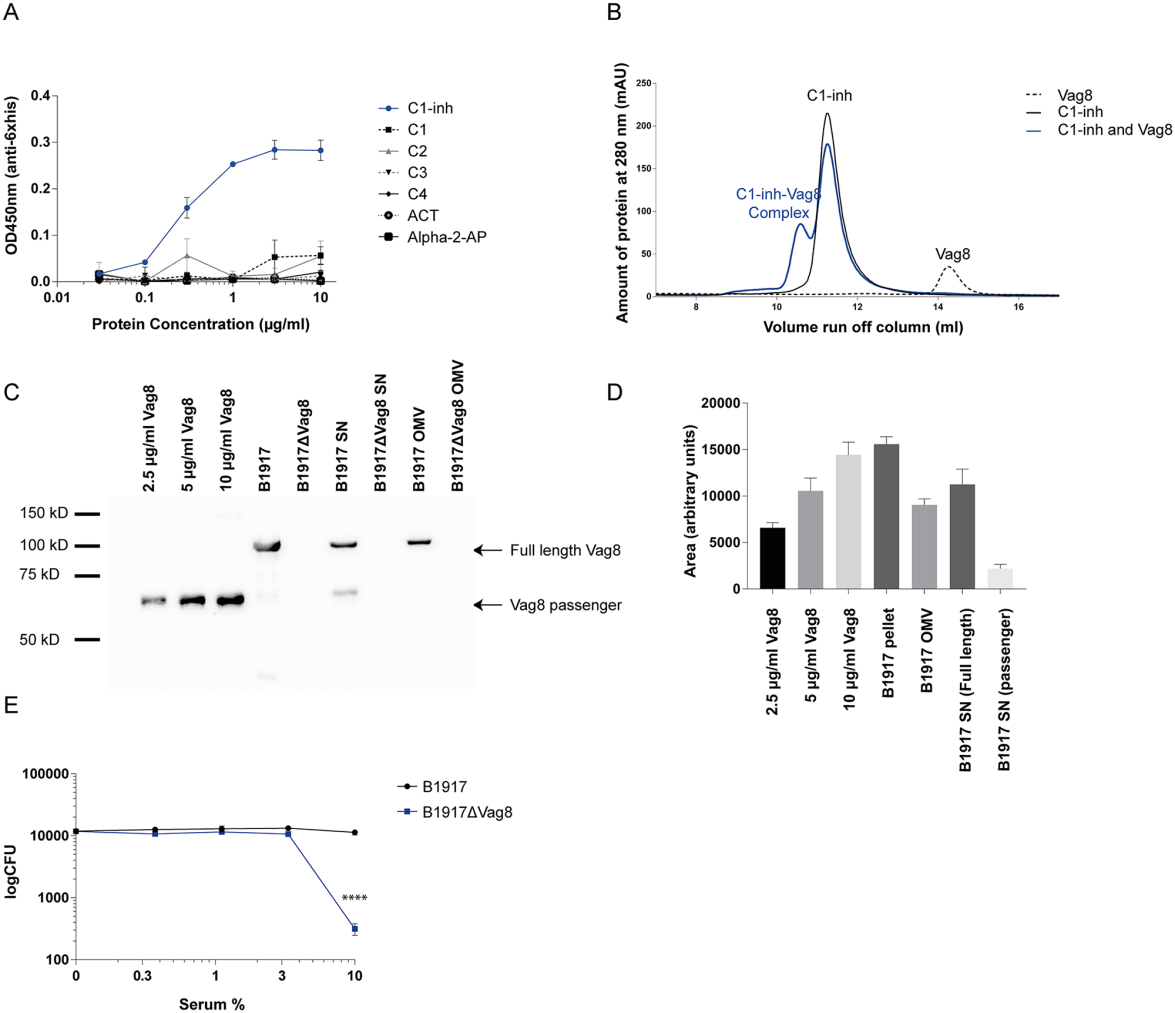
Vag8 binds C1-inh and is involved in serum resistance. (A) Vag8 binds to C1-inh in a dose dependent manner. No binding was observed to the serpins alpha-1-antichymotrypsin and alpha-2-antiplasmin or the complement components C1, C2, C3 and C4 by ELISA. (B) Vag8 forms a stable complex with C1-inh in fluid phase as shown by making use of the gel filtration chromatography method. (C) The successful construction of the *B*. *pertussis* B1917ΔVag8 mutant strain was confirmed by immunoblot. No Vag8 could be detected in the pellet or the bacterial supernatant or OMV’s of B1917ΔVag8. In addition, the *B*. *pertussis* wild type strain B1917 expressed both the full length Vag8 as well as the passenger domain in the supernatant. (D) Using ImageJ, the intensity of the Vag8 bands were semi quantified relative to known concentrations of recombinant Vag8. We show that 10^7^ bacteria of the B1917 parental strain contain 10 μg/ml Vag8 and 10^9^ bacteria secrete 5–10 μg/ml of full length and 1 μg/ml of passenger Vag8. Moreover, 10 μg/ml OMV contains 5 μg/ml of Vag8. (E) The B1917ΔVag8 mutant strain shows increased sensitivity to serum-mediated killing compared to the B1917 parent strain. Data shown in Fig 1A, 1D and 1E represent the mean ± SEM of three separate experiments while Fig 1B and 1C are representative of three separate experiments.

It was previously shown that C1-inh binding to Vag8 of *B*. *pertussis* is linked to serum resistance. However, the molecular mechanism for this serum resistance was not investigated [7]. To study the complement evasion mechanism of Vag8, recombinant Vag8 was produced and circular dichromism spectroscopy was used to confirm correct folding of the protein in β-sheets (S1 Fig). We confirmed that Vag8 can bind to C1-inh using an ELISA based system (Fig 1A). In addition to this previously described observation, we show that Vag8 binding to C1-inh is specific since no binding was detected to either the serpins alpha-1-antichymotrypsin and alpha-2-antiplasmin or the complement components C1, C2, C3 and C4 (Fig 1A). Furthermore, using gel filtration, we show that Vag8 can form a stable complex with C1-inh in fluid phase (Fig 1B) which has not been shown before. The presence of C1-inh and or Vag8 in the appropriate single or complex peaks was confirmed using immunoblotting (S2 Fig).

As described previously, Vag8 is an autotransporter present on the outer membrane of *B*. *pertussis* as well as on OMVs. A previous study was unable to detect the secreted passenger domain of Vag8 indicating that the passenger domain of Vag8 would be retained on the bacterial surface [5]. To study the production of the passenger domain of Vag8 during liquid growth and to verify the involvement of Vag8 in serum resistance of *B*. *pertussis*, we constructed a *vag8* mutant in a B1917 background (further referred to as B1917ΔVag8). B1917 is a recently circulating *B*. *pertussis* strain carrying the *ptxP*3 allele [32]. Analysis of the bacterial pellets, bacterial OMVs and bacterial supernatant by immunoblotting shows detection of full length Vag8 in the bacterial pellets of B1917 parent strain while there is no detectable Vag8 in the pellets of B1917ΔVag8 confirming the successful deletion of *vag8* (Fig 1C). In addition, the supernatant of B1917 shows the presence of full length Vag8, which is most likely present on OMVs, as well as the passenger domain of Vag8 (Fig 1C) which is in contrast to previously published data [5, 6]. ImageJ was used to estimate the amounts of Vag8 present in the different B1917 parent strain fractions relative to known concentrations of recombinant Vag8. We show that 10^7^ bacteria express around 10 μg/ml Vag8, 10^9^ bacteria secrete around 5 μg/ml full length Vag8 and around 1 μg/ml passenger Vag8. Furthermore, 10 μg/ml of OMVs contain 5 μg/ml Vag8 (Fig 1D). Next, the involvement of endogenously secreted Vag8 in serum resistance of *B*. *pertussis* was analysed. As expected, decreased survival of B1917ΔVag8 was observed compared to the parent strain B1917 upon addition of increasing serum concentrations (Fig 1E). In conclusion, we verify that Vag8 binds to C1-inh and we show that this binding is specific and stable in fluid-phase. Next, we verify that bacterial Vag8 expression is involved in serum resistance of *B*. *pertussis*. Furthermore, we show that the Vag8 passenger domain is expressed and secreted by *B*. *pertussis* strain B1917 during liquid growth conditions.

### Vag8 inhibits the CP and LP at the level of C4b and subsequent bacterial killing

**Fig 2:**
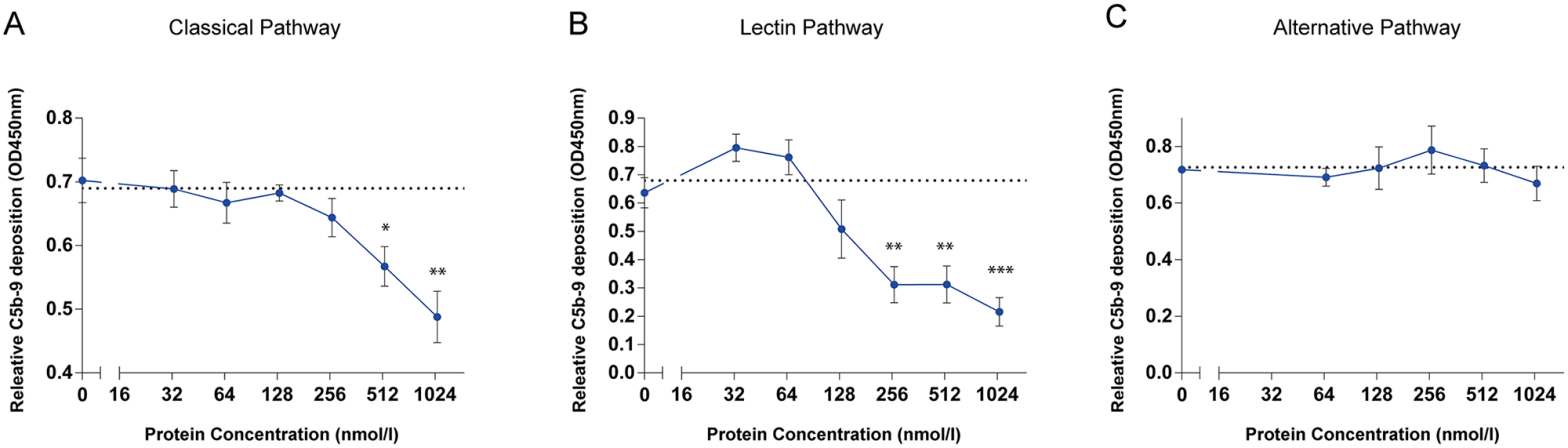
Vag8 inhibits the CP and LP but not the AP. Using complement ELISAs, we show that Vag8 dose dependently inhibits C5b-9 deposition of the (A) CP and (B) LP but not of the (C) AP. The dashed line indicates complement activation in buffer only. Data shown in A-C represent the mean ± SEM of three separate experiments. Significant differences compared to Buffer are indicated with a * * p≤0.05, ** p≤0.01, *** p≤0.001.

To investigate the mechanism of action of Vag8 complement inhibition, the influence of recombinant Vag8 on the three different complement pathways was studied. We investigated the effect of Vag8 on C5b-9 deposition. Fig 2A and 2B show that Vag8 inhibits C5b-9 deposition via the CP and LP in a dose dependent manner, while the AP C5b-9 deposition was unaffected (Fig 2C).

**Fig 3:**
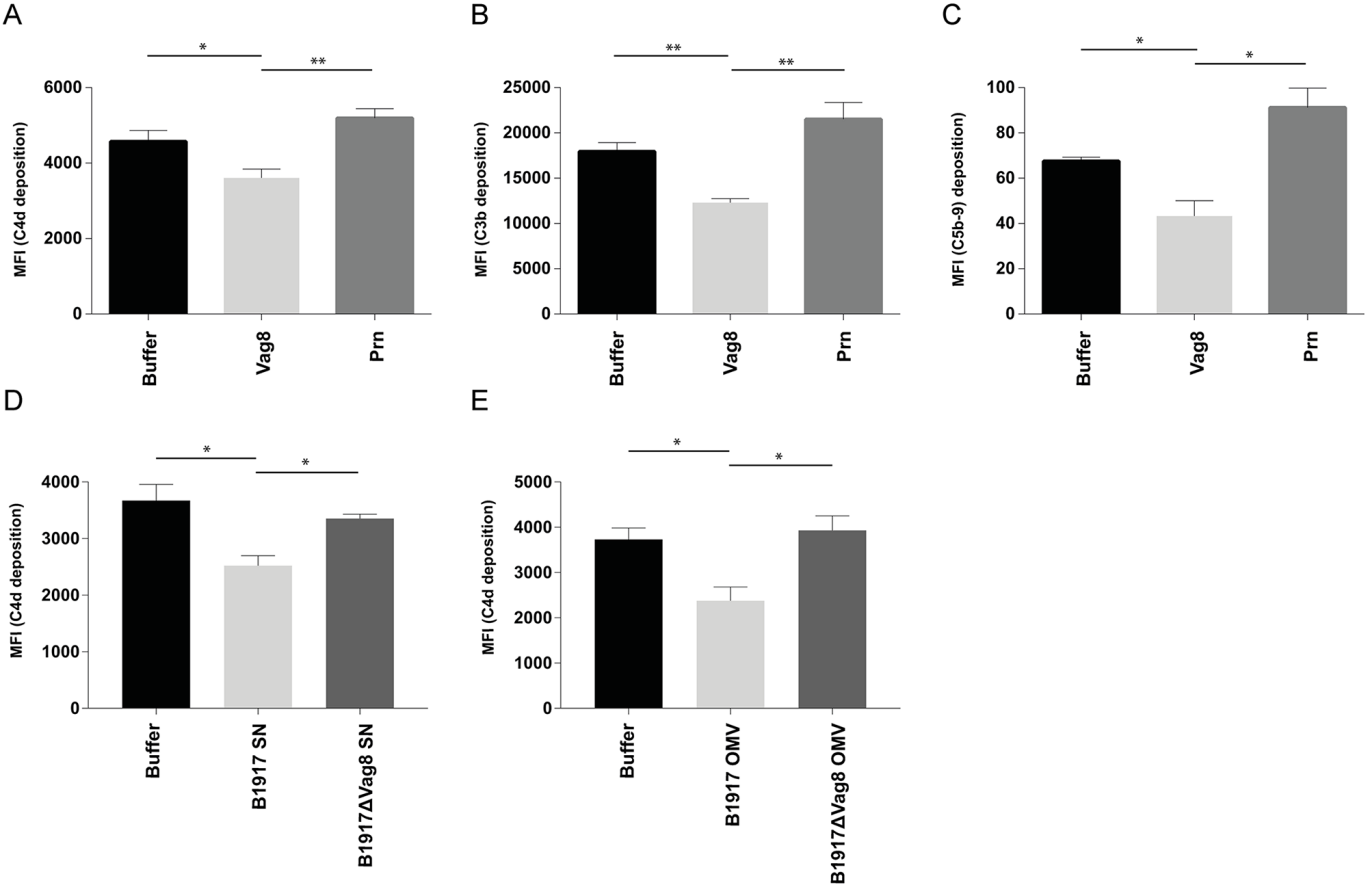
Vag8 inhibits complement deposition on *B*. *pertussis*. The addition of Vag8 to *B*. *pertussis* B1917 opsonized with 1.25% NHS shows decreased deposition of (A) C4b, (B) C3b and (C) C5b-9 compared to buffer or addition of the negative control Prn. Additionally, (D) C4b deposition on B1917 was decreased in the presence of 1.25% NHS and bacterial supernatant of wild type *B*. *pertussis* strain B1917 but not of the B1917ΔVag8 mutant. Moreover, (E) C4b deposition on B1917 was decreased in the presence of 1.25% NHS and OMVs derived from the wild type *B*. *pertussis* strain B1917 but not OMVs of the B1917ΔVag8 mutant. Data shown in A-E represent the mean ± SEM of three separate experiments. * p≤0.05, ** p≤0.01

To assess the effect of Vag8 in a more physiological setting, complement deposition was determined on *B*. *pertussis* strain B1917. Pertactin (Prn), known as a minor adhesin, is another autotransporter protein of *B*. *pertussis* with a structure and size similar to Vag8 [33] and was therefore taken along as a negative control. Bacteria were incubated with 1.25% normal human serum (NHS), where the CP and LP are mainly active, in the presence or absence of Vag8 or Prn. We determined the deposition of C4b, C3b and MAC (C5b-9). C4b is part of the CP and LP C4b2a C3 convertase which is formed upon cleavage of C4 by the C1 and MBL complex proteases C1s and MASP-2 following the recognition of antigen-antibody complexes by the C1 complex or of sugar patterns by the MBL/MASP complex respectively [14]. C3b is subsequently formed upon C3 cleavage by the C3 convertase (C4b2a) and gives rise to the AP C3 convertase (C3bBb) and subsequently to the CP and LP C5 convertase (C4b2aC3b) or AP C5 convertase(C3bBbC3b) eventually resulting in MAC (C5b-9) formation [14]. Flow cytometric data demonstrate that Vag8 inhibits C4b deposition (Fig 3A) on the bacterial membrane as well as subsequent C3b and C5b-9 deposition (Fig 3B and 3C). No complement inhibitory effects of the negative control Prn were observed. To determine whether endogenous Vag8 secreted by *B*. *pertussis* can also mediate the observed decrease in complement deposition, supernatant was collected from the B1917 parent strain and B1917ΔVag8. As shown in Fig 1C, both full length Vag8 and the passenger domain of Vag8 were detected in the supernatant of the B1917 parent strain. We show, that the addition of supernatant of B1917 during opsonisation of *B*. *pertussis* results in significantly decreased C4b deposition compared to addition of supernatant of B1917ΔVag8 (Fig 3D). This suggests that *B*. *pertussis* can make sufficient amounts of Vag8 to inhibit the complement system under physiological growth conditions. Moreover, C4b deposition was significantly decreased in the presence of OMVs derived from the B1917 parent strain compared to incubation with B1917ΔVag8 derived OMVs (Fig 3E).

**Fig 4:**
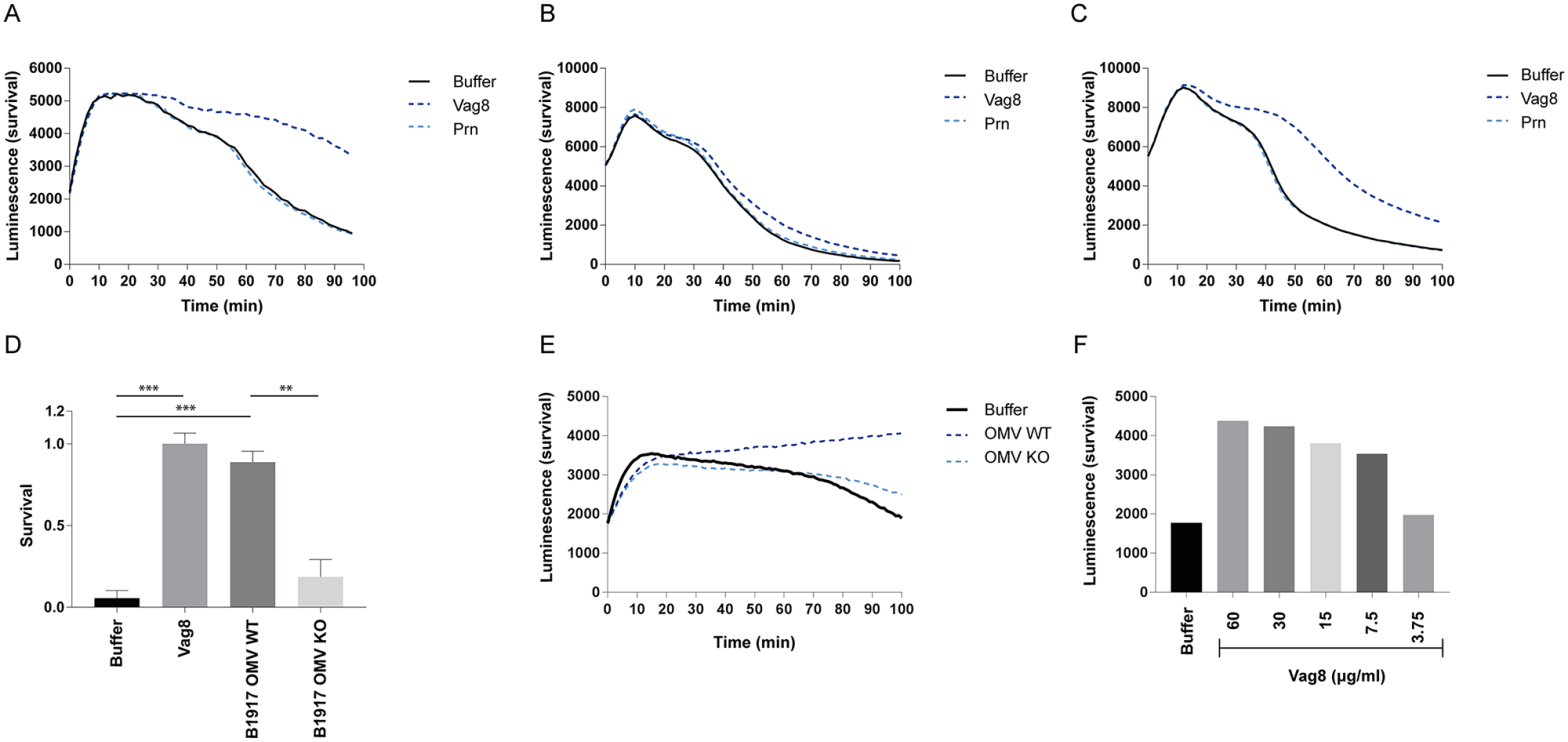
Vag8 inhibits complement-mediated killing of bacteria. (A) Complement-mediated killing of luminescent *E*. *coli* in 1.25% NHS is inhibited in the presence of Vag8 but not Prn. (B) No inhibitory effect on complement-mediated killing was shown for Vag8 in 5% NHS. (C) Vag8 inhibits complement-mediated killing of luminescent *E*. *coli* in 5% fDd serum. (D) B1917 wild type OMVs containing Vag8 inhibit complement-mediated killing of *E*. *coli* using traditional serum killing and CFU counting, as well as of (E) luminescent *E*. *coli*. (F) Complement-mediated killing of luminescent *E*. *coli* is inhibited using 7.5 μg/ml or more Vag8. Data shown in A-C and E-F is a representative figure of three separate experiments. Data shown in D represent the mean ± SEM of three separate experiments. ** p≤0.01, ***≤0.001

Since Vag8 has proven to efficiently inhibit complement deposition on a bacterial membrane, we subsequently studied the effect of Vag8 on bacterial killing using a luminescent Gram-negative *Escherichia coli* [34], as well as traditional plating. Survival of the luminescent *E*. *coli* in 1.25% NHS with or without Vag8 and the negative control Prn was determined by monitoring the luminescent signal over time. A decreased luminescent signal, indicating bacterial killing, is observed over time in the presence of buffer or Prn, while upon addition of Vag8 this killing is strongly inhibited (Fig 4A). The inhibitory effect of Vag8 was lost in the presence of higher serum concentrations (5% NHS) (Fig 4B), probably due to the involvement of the AP which Vag8 is not able to inhibit (Fig 2C). In line with this hypothesis, the inhibitory effect of Vag8 is indeed clearly visible in the presence of 5% factor D deficient (fDd) serum in which the AP can no longer function (Fig 4C) again showing that Vag8 inhibits the CP and the LP of the complement system. Using traditional serum killing, we also show clear inhibition of complement-mediated killing by recombinant Vag8 using fDd serum (Fig 4D). Subsequently, we assessed the effect of endogenous Vag8 on bacterial killing by incubating *E*. *coli* with OMVs either derived from the B1917 parent or B1917ΔVag8 strain and human serum. We show that OMVs containing Vag8 significantly decrease complement-mediated killing of *E*. *coli* using both colony forming units (CFUs) (Fig 4D) and luminescence (Fig 4E) as a readout. To determine the minimum amount of Vag8 needed to inhibit complement-mediated killing via the CP and LP, the serum killing experiment using luminescent *E*. *coli* was repeated using a concentration range of Vag8 and 1.25% fDd serum. We show that 7.5 μg/ml of Vag8 is sufficient to inhibit complement-mediated killing (Fig 4F). Collectively, these data demonstrate that recombinant and endogenously secreted Vag8 inhibits the deposition of complement effector molecules on the bacterial surface and thereby prevents subsequent bacterial killing via the CP and LP.

### Vag8 prevents binding of C1s, C1r and MASP-2 to C1-inh

**Fig 5:**
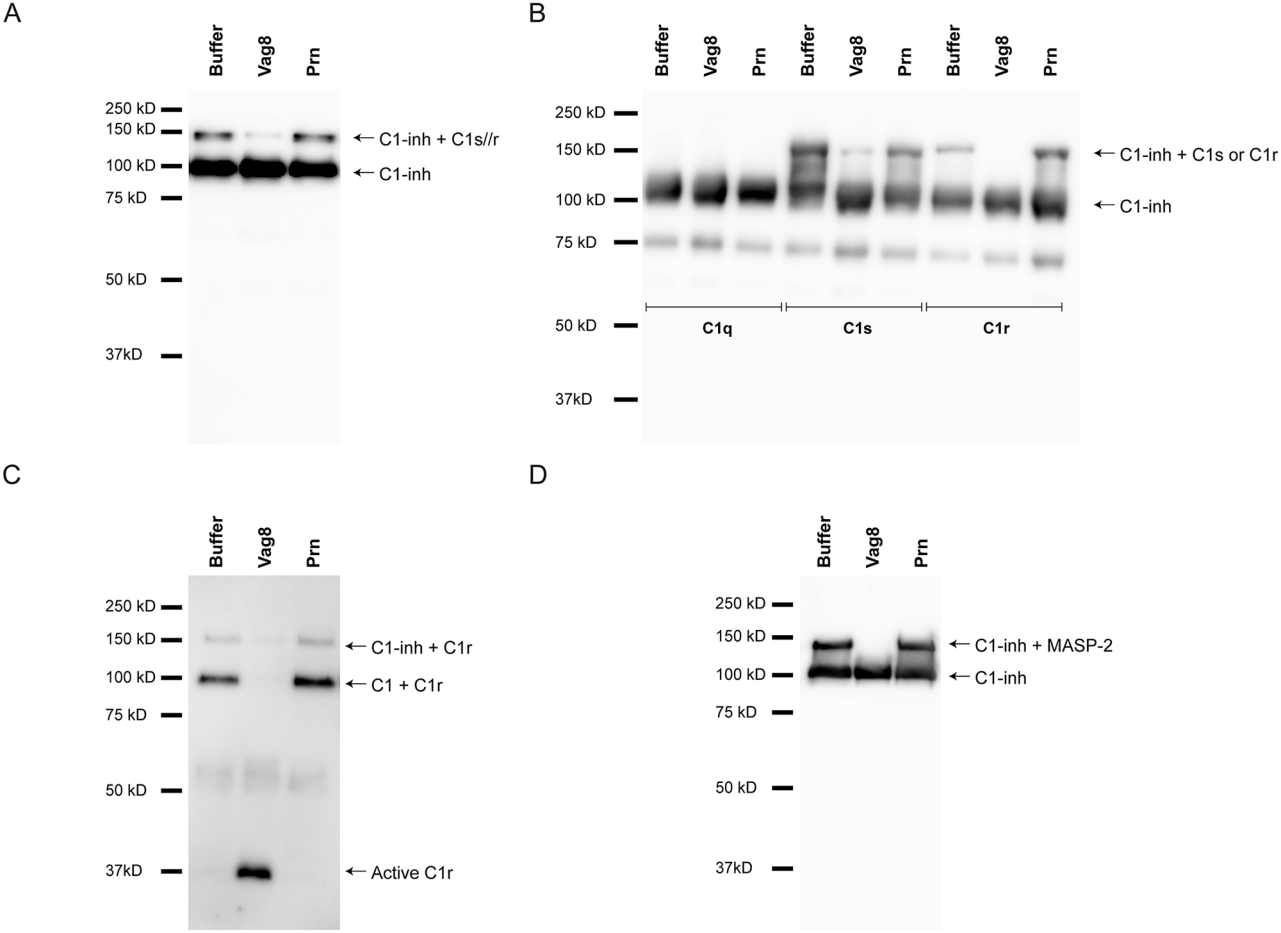
Vag8 interferes with the binding of C1s, C1r and MASP-2 with C1-inh. (A) *B*. *pertussis* strain B1917 was opsonized with 1.25% NHS with or without Vag8 or Prn. Supernatant was analysed using anti C1-inh. The presence of Vag8 results in a decreased signal of the ~150 kDa band. (B) In a purified system consisting of C1q, C1s or C1r with C1-inh in the presence or absence of Vag8 or Prn, we show, using anti-C1-inh, that C1s and C1r, but not C1q, bind to C1-inh and that this binding is inhibited by the addition of Vag8 but not Prn. (C) C1r was detected using anti-C1r. The presence of C1r bound to C1 and C1-inh is inhibited in the presence of Vag8 but not Prn. Additionally, we show the presence of active C1r in the presence of Vag8. (D) Purified MASP-2 was incubated with C1-inh in the presence of Vag8 or Prn and analyzed using anti-C1-inh. We show inhibition of MASP-2 binding to C1-inh in the presence of Vag8, but not Prn. Immunoblots are representative of three separate experiments.

As previously described, Vag8 binds to C1-inh (Fig 1A and [7]) and prevents complement deposition on the bacterial surface (Fig 3A–3E). C1-inh belongs to the serine protease inhibitor superfamily and can inactivate and dissociate the proteases of the C1 and MBL/MASP complex [35]. The C1 complex consists of C1q and a dimer of the proteases C1s and a dimer of the protease C1r, whereas the MBL/MASP complex contains one MASP-1 and one MASP-2 homodimer [15, 16, 18, 20, 21]. Inactivation of C1s, C1r, MASP-1 and MASP-2 from their respective complexes occurs in a two-step manner. First, the reactive center loop mimics the protease’s substrate. This loop is exposed at the surface of C1-inh and is recognized by the target protease. Subsequently, the target proteases cleave the center loop and trigger a molecular rearrangement. This results in the formation of a covalent bond between C1-inh and the active site serine of the protease, inactivating the latter [36]. To investigate the effect of Vag8 binding to C1-inh during bacterial opsonisation, we determined the levels of C1-inh in supernatants taken from *B*. *pertussis* incubated with NHS in the presence or absence of Vag8 or Prn using immunoblotting. We detected C1-inh (approximately 110 kDa) and C1-inh probably bound to C1s and/or C1r (approximately 150 kDa) (Fig 5A) [37]. Notably, upon the addition of Vag8 this 150 kDa band was strongly reduced indicating that Vag8 binding to C1-inh interferes with this interaction. We next used a purified system to investigate whether the observed 150 kDa band indeed corresponded to C1-inh binding to active C1s or C1r and whether Vag8 was responsible for the loss of protease binding to C1-inh. C1-inh was either incubated with molecular ratios of C1q, C1s or C1r in the presence or absence of Vag8 or Prn. As expected, both C1s and C1r bound to C1-inh while C1q did not (Fig 5B). Moreover, we observed a clear inhibition of C1s binding to C1-inh and a full inhibition of C1r binding to C1-inh by addition of Vag8 (Fig 5B). The negative control Prn did not show inhibitory properties. To assess the ability of Vag8 to prevent C1r and C1s binding to C1-inh in a more physiological setting, we analyzed C1r and C1s binding to C1-inh and C1 in the supernatant of bacteria opsonized with NHS in the presence or absence of Vag8 or Prn. We confirm the loss of C1r binding to C1-inh and show the loss of C1r binding to C1 in the presence of Vag8 (Fig 5C). Moreover, the active form of C1r can be detected in the presence of Vag8 as can be expected in the absence of bound C1-inh. Detection of C1s yielded similar results (S3 Fig). Vag8 inhibits both the CP and the LP, hence, the effect of Vag8 on the binding of MASP-2 to C1-inh was additionally analysed in a purified system. Similar to the results of C1s and C1r binding to C1-inh, we show that the binding of MASP-2 to C1-inh is lost in the presence of Vag8 but not in the presence of Prn (Fig 5D). In summary, we show that Vag8 effectively prevents C1s, C1r and MASP-2 binding to C1-inh.

### Vag8 mediates consumption of the complement components C4 and C2

**Fig 6:**
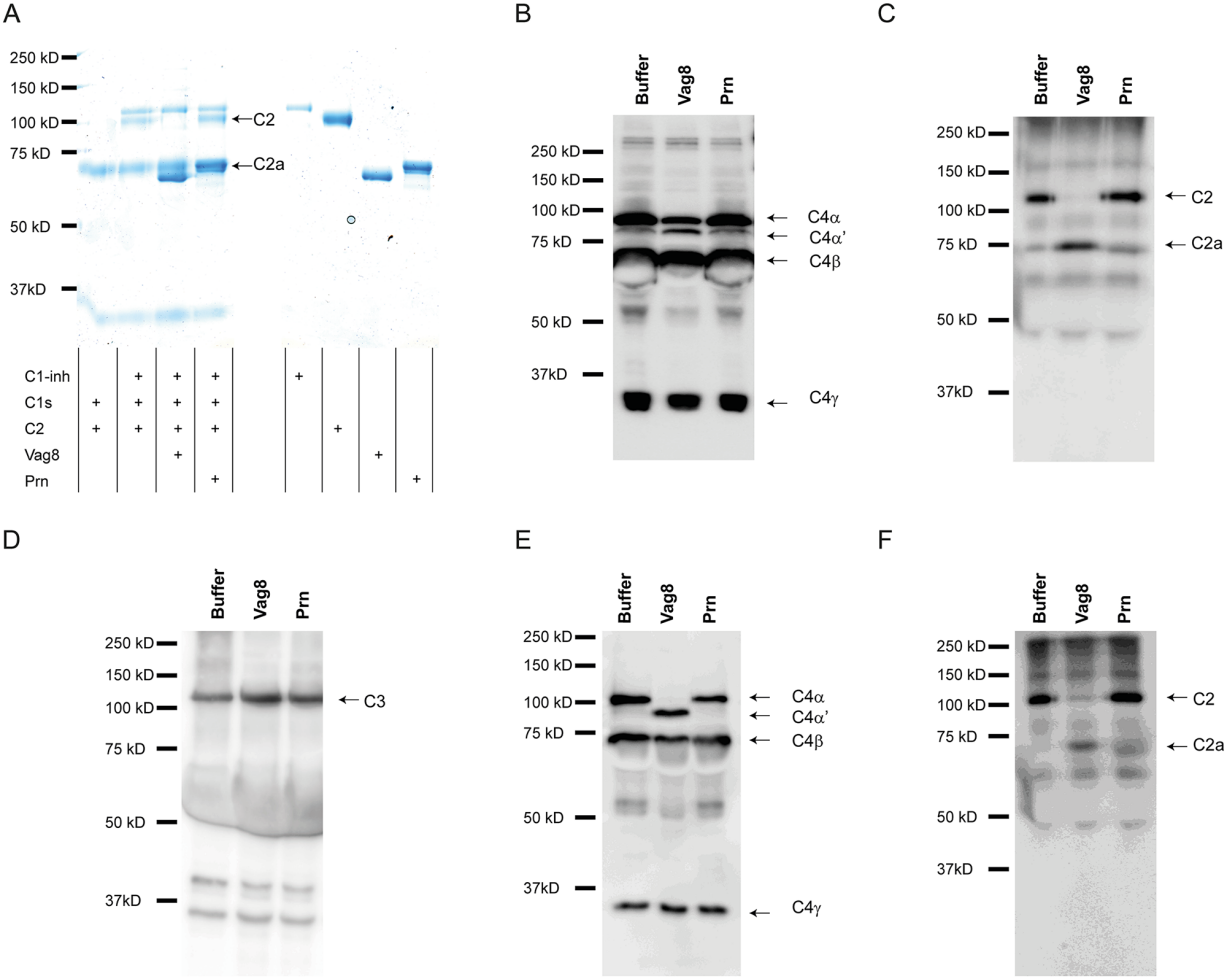
Vag8 mediates degradation of C4 and C2. (A) Purified C1s and C2 were incubated alone, or with C1-inh, in the presence of Vag8 or Prn and visualized using Instant Blue. Increased C2 cleavage is shown in the presence of Vag8. *B*. *pertussis* B1917 was opsonized with 1.25% NHS with or without Vag8 or Prn. Supernatant was analysed by immunoblot and shows cleavage of (B) C4, (C) C2, but not (D) C3 in the presence of Vag8 compared to control situation. Incubation of 1.25% NHS alone with or without Vag8 or Prn shows increased cleavage of (E) C4 and (F) C2 in the presence of Vag8. All figures are representative for three separate experiments.

**Fig 7:**
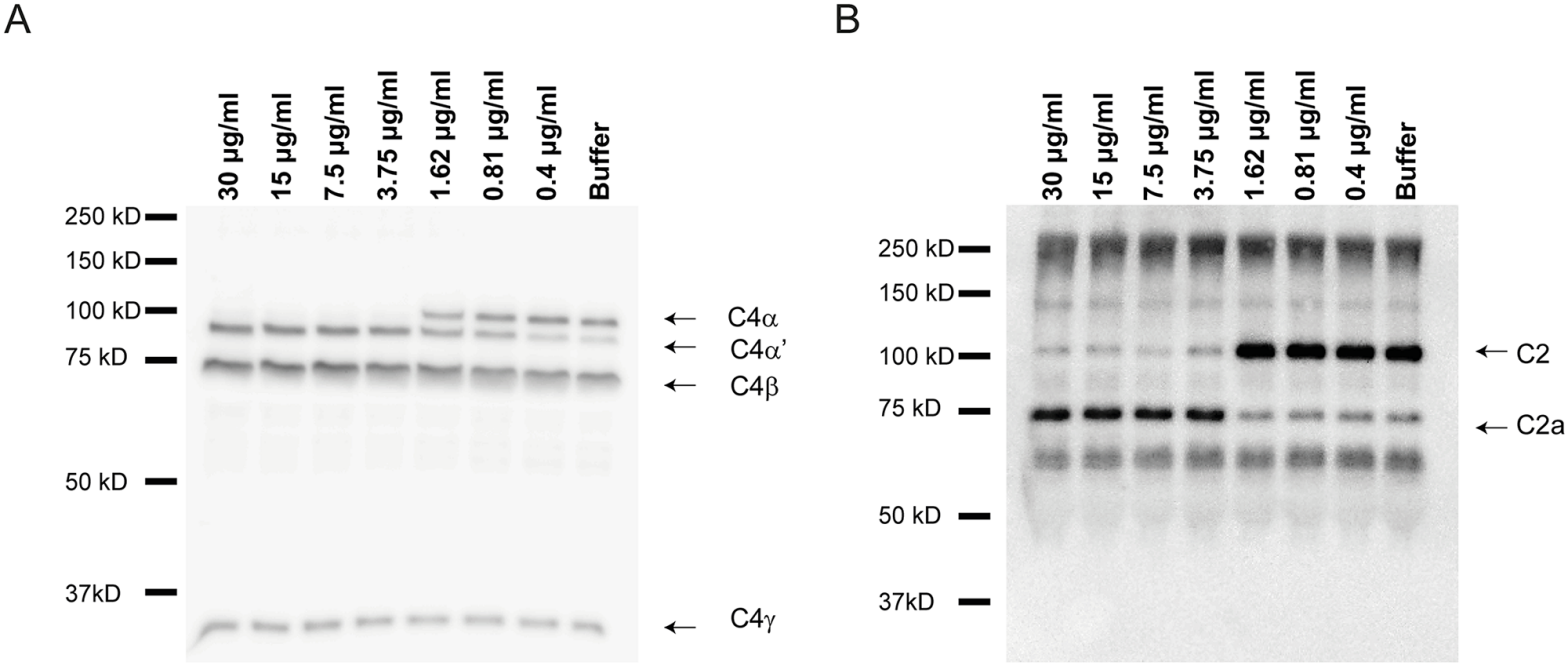
Degradation of C4 and C2 by Vag8. Incubation of NHS with different concentrations of Vag8 shows complete cleavage of (A) C4 and (B) C2 in the presence of *B*. *pertussis* B1917 using 3.75 μg/ml or more Vag8. All figures are representative for three separate experiments.

During complement activation, C2 is cleaved by C1s, MASP-1 and MASP-2, whereas C4 is cleaved by C1s and MASP-2 only. This cleavage is inhibited by C1-inh [14]. We investigated the effect of Vag8 binding to C1-inh on C2 and C4 cleavage in a purified system or in the presence of *B*. *pertussis* strain B1917. Addition of Vag8 to a purified system consisting of C1-inh, C1s and C2, results in increased cleavage of C2 (Fig 6A). This increased cleavage was not observed by the addition of Prn. Vag8 alone was unable to cleave C2 (S4 Fig). To determine whether this phenomenon also occurs in the presence of bacteria, we incubated *B*. *pertussis* strain B1917 with NHS in the presence or absence of Vag8 or Prn. Supernatants were collected and C2, C4 and C3 cleavage was assessed. Increased cleavage of C4 (Fig 6B) and C2 (Fig 6C) in the supernatant of bacteria incubated with NHS and Vag8 was observed compared to the control situations. C3 cleavage in the presence of Vag8 remained comparable to bacteria alone or in the presence of Prn (Fig 6D). Furthermore, to assess whether addition of Vag8 to NHS alone leads to increased C4 and C2 cleavage, we analysed the cleavage of C4 and C2 in NHS upon the addition of Vag8 or Prn compared to NHS with buffer. Fig 6E and 6F show that addition of Vag8 to NHS induced complete C4 and C2 consumption. To determine the minimum amount of Vag8 needed to fully cleave C4 and C2, the experiments mentioned above were repeated using a concentration range of Vag8. We show that 3.75 μg/ml of Vag8 is sufficient to lead to the complete degradation of C4 (Fig 7A) and C2 (Fig 7B) in the presence of bacteria. Moreover, incubation of NHS alone with 3.75 μg/ml or 7.5 μg/ml Vag8 results in full cleavage of C4 or C2 respectively (S5 Fig). In conclusion, these results indicate that low amounts of Vag8 binding to C1-inh, results in the consumption of C4 and C2 away from the bacterial surface and in NHS alone, whereas C3 cleavage remained constant.

## Discussion

Whooping cough, the highly infectious respiratory disease caused by the human-specific *B*. *pertussis*, has been re-emerging in the past decades despite a high vaccine coverage [38]. The necessity of a novel improved vaccine has become evident and complement evasion molecules have been proposed as potential vaccine candidates [12]. The autotransporter Vag8 is one of these complement evasion molecules. Interestingly, it is also expressed more by emerging strains compared to older strains [8, 9]. Due to the presence of Vag8 on OMVs, which have been proposed as novel pertussis vaccines [6], as well as its presence on the live attenuated pertussis vaccine BPZE1 [39] it is of great importance to unravel the complement inhibitory functions of Vag8 since this might have negative effects upon vaccination. The C-terminus of Vag8 has high sequence identity to other *B*. *pertussis* autotransporter proteins including Prn [40]. Previous data indicated that unlike most autotransporters [41, 42], the passenger domain of Vag8 would not be cleaved [5]. Recently, Vag8 was described to be part of the *B*. *pertussis* secretome, with secreted levels being comparable to Prn and even higher than Ptx [43]. However, this recent paper does not specify whether the full length Vag8 was secreted or the passenger alone. Here, we show the presence of the passenger domain of Vag8 in the supernatant of *B*. *pertussis* strain B1917 with the correct predicted molecular weight. In addition, we also detect the full length Vag8 in the supernatant. This full length Vag8 is most probably present on OMVs. OMVs have previously been shown to be present in the supernatant of *B*. *pertussis* grown in Stainer and Scholte (SS) medium as well as to contain Vag8 [6, 44].

Our results show that Vag8 of *B*. *pertussis* inhibits complement activation via the CP and LP, but not the AP. Multiple bacteria can inhibit the initiation of the CP by directly targeting the C1 complex. *Staphylococcus aureus*, for example, expresses microbial surface components that bind to the stalk region of C1q disrupting proper C1 complex formation [45], whereas *Borrelia burgdorferi* expresses BBK32 which prevents the autoactivation of C1r and subsequent C4 and C2 cleavage [46]. Alternatively, the CP, but also the LP, can be inhibited by hijacking the host regulatory proteins C1-inh and C4b binding protein (C4bp) [28]. C1-inh prevents the activation of the CP and LP by inhibiting C1r and C1s as well as MASP-1 and MASP-2 activity respectively [16]. C1-inh is, for example, targeted by the outer membrane lipoprotein CihC of *Borrelia recurrentis* [47]. Data suggests that the interaction between C1-inh and this protein results in the recruitment of C1-inh to the bacterial surface leading to the local inactivation of C1s and C1r and thus complement inactivation [47]. Similar to other bacteria, *B*. *pertussis* is able to evade the complement system. Vag8, as well as Bordetella resistance to killing (BrkA), *B*. *pertussis* autotransporter protein C (BapC) and filamentous hemagglutinin (FHA), have been implicated to be involved in complement evasion by *B*. *pertussis* [7, 48–50]. FHA has been shown to bind C4bp, however, a physiological role for this binding remains to be investigated since a mutant strain lacking FHA was equally resistant to complement killing compared to the wild type strain [51]. Furthermore, the complement evasion mechanism of BrkA [48] and BapC [50] remain not fully understood. Vag8 of *B*. *pertussis* has previously been shown to bind C1-inh [7] and it was speculated that Vag8 would bind C1-inh to the surface of *B*. *pertussis*, thereby mediating serum resistance.

Here, we have unraveled the molecular mechanism by which Vag8 inhibits complement-mediated lysis of *B*. *pertussis*. We show that Vag8 mediates complement evasion of the CP and LP via a previously undescribed mechanism. This novel complement evasion strategy involves the binding of Vag8 to C1-inh away from the bacterial surface. Binding of Vag8 to C1-inh interferes with the interaction of C1s, C1r and MASP-2 with C1-inh resulting in the fluid phase cleavage and consumption of C4 as well as C2. C4 and C2 are the two most important proteins for activation of the CP and the LP. We show that only 3.75 μg/ml of Vag8 is needed to result in complete degradation of C4 and C2 in the presence of bacteria (Fig 6F and 6G) and that at least this amount of Vag8 is secreted by the bacteria and is present on OMVs (Fig 1D). Next, we have shown that only 7.5 μg/ml of Vag8 is needed to prevent complement-mediated bacterial killing. In addition, several studies have shown that Vag8 is highly expressed by *B*. *pertussis in vitro* either under physiological conditions in several liquid media including Thalen-IJssel (THIJS) and SS media or during biofilm formation [5, 43, 52, 53]. Upon analysing the proteome of *B*. *pertussis*, the levels of Vag8 expression as well as secretion were comparable to Prn and even higher than Ptx [11, 43]. Furthermore, transcriptional analysis of *B*. *pertussis* shows that Vag8 is expressed *in vivo* upon infection [11, 54]. Pulmonary and systemic antibodies directed against Vag8 could be detected following a pertussis challenge [55]. Also human pertussis infection gives rise to antibodies directed against Vag8 indicating expression during infection [56]. Taken together, Vag8 is highly expressed *in vitro* and *in vivo* and a low amount of Vag8 is needed to prevent complement-mediated bacterial killing.

**Fig 8:**
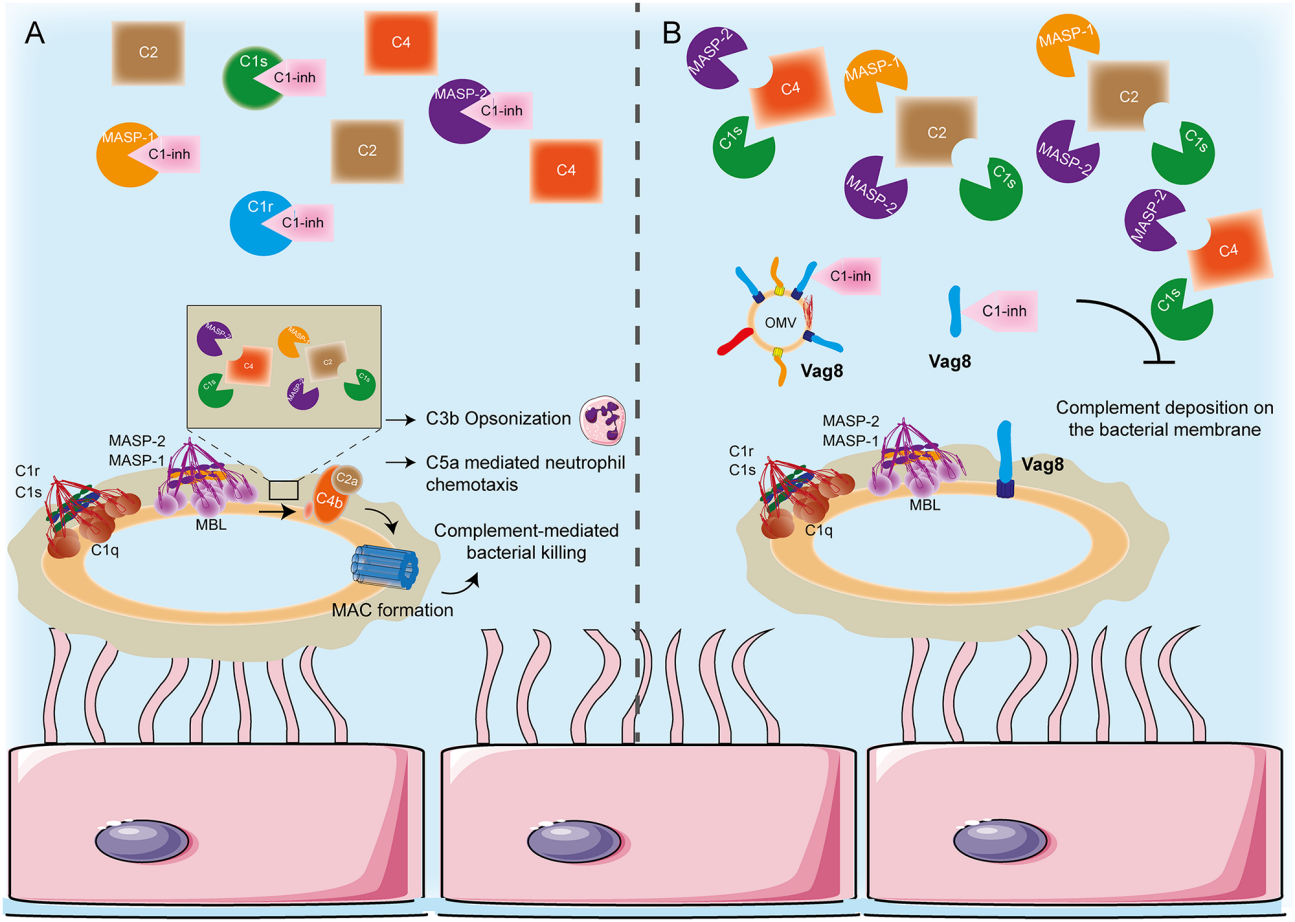
Proposed mechanism for Vag8 mediated complement evasion. (A) In the absence of Vag8, C1 and MBL/MASP complexes are formed on the bacterial surface which results in the activation of the CP and LP proteases. These proteases cleave C4 and C2 and give rise to the C3 convertase (C4bC2a). Any C1s, C1r, MASP-1 or MASP-2 not part of a bacterial surface bound complex remains inactive by association with C1-inh and hence free intact C4 and C2 is available for recruitment upon complement activation on bacteria. (B) In the presence of Vag8, either the secreted passenger or as part of an OMV, the interaction between C1s, C1r, MASP-1 or MASP-2 and C1-inh is interrupted as Vag8 hijacks this inhibitor. This results in the presence of active proteases in the bacterial environment which cleave and hence deplete the free C4 and C2. The lack of C4 and C2 to be cleaved and deposited on the bacterial membrane upon recognition of *B pertussis* by C1 and MBL complexes will lead to decreased complement deposition on the surface of *B*. *pertussis* and subsequent bacterial killing. Epithelial cells were adapted from Servier Medical Art, provided by Servier under a CC-BY 3.0 license (available at: http://www.servier.com/powerpoint-image-bank).

In line with our observations, it has been shown that in order for Vag8 to bind C1-inh the active serpin-domain conformation of C1-inh is required [31] which may explain the loss of C1s, C1r and MASP-2 binding to C1-inh that we observe in this study. Moreover, C1-inh deficiency in humans result in a disease called hereditary angioedema which is in part characterized by low serum levels of C4, similar to what we observe in the presence of Vag8 [57]. Taken together, we show that secreted Vag8, either present on OMVs or as passenger only, leads to C4 and C2 consumption away from the bacterial surface. This results in inhibition of complement deposition on the bacterial membrane and complement-mediated lysis (Fig 8). While we cannot exclude a function for C1-inh binding to the surface of *B*. *pertussis*, we hypothesise that Vag8 binding to C1-inh away from the bacterial surface is very important for complement evasion. Complement consumption by a bacterial pathogen is not a new phenomenon since *S*. *aureus* staphylococcal immunoglobulin-binding as well as the metalloprotease aureolysin mediate C3 consumption subsequently inhibiting not only the CP and LP but also the AP [58, 59]. Vag8 induced complement consumption is solely detected at the level of C4 and C2, as C3 levels and cleavage were comparable in the presence or absence of Vag8, and hence is limited to the CP and LP, the most important pathways for eradication of *B*. *pertussis* [60].

In the light of pertussis resurgence, novel vaccine components and strategies are being explored including the use of Vag8 [6, 12]. In a murine experimental model, vaccination with recombinant Vag8, giving rise to antibodies directed against Vag8, resulted in a nearly 10-fold reduction of bacterial load following a challenge [11]. In line with these findings, it was shown that the humoral response upon whole cell pertussis vaccination, but also natural infection, was mainly characterized by antibodies raised against Vag8 [6, 55]. As previously mentioned, 34% of the *B*. *pertussis* OMV protein content is Vag8 which might be even more according to Fig 1D. Therefore, it is not surprising that vaccination with OMVs also resulted in the production of antibodies directed against Vag8 [6]. The importance of these antibodies in a protective immune response might lie in the inhibition of the observed complement consumption here described. Nonetheless, it is important to keep in mind that vaccination with the passenger domain of Vag8, pertussis OMV’s and the life attenuated pertussis vaccine BPZE1 that contain Vag8 could induce side effects as a result of local complement depletion and a potential vaccine antigen might have to be modified to avoid this. Blocking antibodies directed against Vag8 would render the bacteria vulnerable for complement-mediated killing hence resulting in a quicker clearance of the bacteria from the respiratory tract preventing successful infection.

In conclusion, we have unravelled the molecular mechanism of Vag8, a potent complement inhibitor secreted by *B*. *pertussis*. With this molecular mechanism more insight is gained into the host-pathogen interaction, which could be used to improve the current pertussis vaccines.

## Materials and methods

### Bacterial strains and growth conditions

All bacterial strains used in this study are shown in Table 1. *B*. *pertussis* strains were grown at 35°C, 5% CO_2_ on Bordet Gengou (BG) plates containing glycerol and 15% defibrinated sheep blood (BD Biosciences). After three to five days of culture, the bacteria were collected in PBS (Gibco), and the optical density (OD) was measured at 600 nm. For liquid growth, *B*. *pertussis* was first grown on plate as described above, subsequently the bacteria were suspended in chemically defined THIJS medium [61] with a start OD of 0.05 and grown ON at 35°C shaking at 130 rpm. *E*. *coli* was grown on LB agar plates or in LB broth with appropriate antibiotics over night at 37°C.

**Table 1:** Characteristics of strains used in this study.

Bacteria	Strain	Plasmid or deletion	Antibiotica	Reference
***B***. ***pertussis***	B1917	Streptomycin-resistant derivative		This study
	B1917	ΔVag8	KAN 50 μg/ml	This study
***E***. ***coli***	Top10f	pGEM Vag8::KAN	KAN 50 μg/ml/ AMP 100 μg/ml	This study
	SM10		KAN 50 μg/ml	[62]
	Top10F	pSS1129(pertussis cloning vector)	Amp 100 μg/ml / Gm 10 μg/ml	[62]
	DH5a	NM522 (kan cassette)	KAN 50 μg/ml	[63]
	MG1655	WT		[64]
	MG1655	pUC18-mini-Tn7T-Gm-lux	Amp 100 μg/ml	[64]
	Top10F1	pRSETb-Vag8	Amp 100 μg/ml	This study
	BL21	pRSETb-Vag8	Amp 100 μg/ml	This study
	Top10F	pGEM-T::Vag8	Amp 100 μg/ml	This study
	Top10F	pGEM-T::Vag8::KAN	Amp 100 μg/ml/ 50 μg/ml	This study
	Top10F	pSS1299::Vag8::KAN	Amp 100 μg/ml/ 50 μg/ml	This study

### Recombinant production of histidine-tagged Vag8 passenger domain and MASP-2

The passenger domain of Vag8 (Val40- Leu610) was cloned from *B*. *pertussis* strain B1917 (accession number: CP009751) using primers listed in Table 2 (Vag8P-Forward and Vag8P-Reverse). The PCR fragment containing a BamHI and NotI restriction site was cloned in pRSETb N-his as described previously [65] with minor modifications. In short, proteins were expressed using a slightly modified pRSET-B vector, adding a non-cleavable 6 residue HIS-tag (MHHHHHHGS) to the N-terminus of the protein. The sequenced plasmid, pRSETB:Vag8 was transformed into *E*. *coli* C41 (DE3) (Lucigen). A single colony was picked and grown in LB broth to an OD660 of 0.6. Expression was induced by addition of 1 mM Isopropyl β-d-1-iogalactopyranoside (IPTG). The histidine-tagged Vag8 passenger was purified under denaturing condition using a HiTrap chelating HP column (GE healthcare life sciences) and eluted using an imidazole gradient. Vag8 passenger was refolded as described previously [7]. Briefly, urea was removed by dialysis to PBS. The correct folding of the passenger was assessed using circular dichroism spectroscopy [66]. MASP-2 was cloned using primers listed in Table 2 (MASP2-forward and MASP2-reverse). The PCR fragment containing a XbaI and EcoRI restriction site was cloned in pRSETb N-his as described above. The sequenced plasmid, pRSETB:MASP-2 was transformed into *E*. *coli* BL21 and expression and purification of MASP-2 was performed as described above.

**Table 2:** Primers used in this study.

Primer name	DNA sequence 5’-3’ (restriction sites underlined)
**Vag8P-Forward**	ATATGGATCCGTCACGGCAGCGCAGCG
**Vag8P-Reverse**	ATATGCGGCCGCCTACAACTCGTTGGTCGGC
**Vag8_1_F**	ATATGGATCCGGTTTAAAACGTCGTGAGACA
**Vag8_1_R**	ATATGTCGACCTCAACACCTCTTGGCTAGAA
**Vag8_2_F**	ATATGTCGACCCGCGCGCCGCCGGCGTCGCTCA
**Vag8_2_R**	AATTATATTCTAGAAGCAACTCGAAAAATGGCGCG
**MASP2-Forward**	GCTCTAGAAATAATTTTGTTTAACTTTAAGAAGGAG
**MASP2-Reverse**	GGAATTCCTTATGTGCTCGTGTAGTGGATCTT

### Generation of *B*. *pertussis* Vag8 knock-out strain

The construction of a Vag8 knock-out strain was performed as previously described [67]. Briefly, a fragment of 1000 base pairs downstream *vag8* was amplified using primers Vag8_1_F and Vag8_1_R and a fragment of 887 base pairs upstream of *vag8* was amplified using primers Vag8_2_F and Vag8_2_R (primers are listed in Table 2). The DNA was cloned into plasmid pGEM-T (according to the manufacturer's instructions) (Promega) resulting in the plasmid pGEM-T::Vag8. Subsequently, a kanamycin resistance gene cassette was cloned into the SalI restriction site resulting in pGEM-T::Vag8::KAN. Then, the Vag8::KAN construct was cloned into pSS1299 [62] resulting in pSS1299::Vag8::KAN and subsequently transformed in *E*. *coli* strain SM10. The latter plasmid was used to replace *vag8* by the *kan* gene into B1917 by allelic exchange [62]. Correct insertion was analysed by PCR and loss of Vag8 expression was confirmed by immunoblotting as described below using 1μg/ml of polyclonal rabbit anti-Vag8 antibody. The Vag8 antibody was produced by Genscript via their New PolyExpress basic package using the following peptide: CGNMGGRVDAGARQY (GenScript).

### Collection of bacterial supernatant and OMVs

*B*. *pertussis* wild type B1917 or the isogenic B1917ΔVag8 mutant were grown over night in THIJS medium and the supernatant was obtained by centrifugation of the bacterial suspension at 2000g for 10 minutes at room temperature. The supernatant was subsequently filtered over a 0.22 μm filter (Millipore). Bacterial supernatant was concentrated 50x for the complement deposition assays or 100x for immunoblot analysis using an Amicon Ultra-15 centrifulgal filter units with Ultracel-50 membrane (Millipore). OMVs were prepared as previously described [68, 69]. The presence or absence of Vag8 in the supernatants and OMVs was visualized by immunoblotting using 1 μg/ml polyclonal rabbit anti-Vag8 antibody as described below.

### Ethics

The study was conducted using blood donation from healthy adults for serum collection. The study was conducted according to the principles expressed in the Declaration of Helsinki and written informed consent was obtained from all blood donors before collection and anonymous use of their samples. Approval was obtained from the medical ethics committee of the UMC Utrecht.

### Serum

NHS was prepared by drawing blood from 20 healthy volunteers. Blood was collected in glass vacutainers (BD biosciences) and allowed to clot for 15 min at room temperature. Serum was collected after centrifugation for 10 min at 1000g at 4°C, pooled and subsequently stored at −80°C. The fDd serum was obtained from Complement Technology.

### Complement-mediated killing of *B*. *pertussis* and *E*. *coli*

To assess complement-mediated killing of *B*. *pertussis* strain B1917 and B1917ΔVag8, 10^5^ live bacteria taken from BG agar plates were incubated with different concentrations of NHS (10–0%, 3-fold) for 60 minutes at 37°C, 5% CO_2_ in 96-well round bottom plates in DMEM medium (Gibco). Bacteria were subsequently plated in duplicate on BG agar plates and CFUs were counted on day four and the log of the counted CFUs was graphically displayed. To determine bacterial killing, *E*. *coli* strain MG1655 lux genes was used (as described by [34]) which is luminescent when metabolically active. The bacteria were grown over night in liquid medium and subsequently diluted to reach mid-exponential phase (OD660 0.5). Bacteria were washed in RPMI supplemented with 0.3% HSA (RPMI-HSA) and diluted to an OD660 of 0.1 after which 1.25 or 5% NHS or fDd serum and 60 μg/ml Vag8, 60 μg/ml Prn, 30 μg/ml OMVs derived from B1917 or B1917ΔVag8 or buffer were added. Luminescence was monitored over time using the Clariostar (BMGlabtech). To determine bacterial killing of *E*. *coli* strain MG1655, the bacteria were grown over night in liquid medium and subsequently diluted to reach mid-exponential phase (OD660 0.5). Subsequently bacteria were diluted to 2 x 10^4^ bacteria per ml and incubated with 1% of fDd serum and 60 μg/ml Vag8, 30 μg/ml of B1917 or B1917ΔVag8 derived OMVs. Bacteria were subsequently plated in duplicate on LB agar plates overnight and (CFU) were counted, the log of the counted CFUs was graphically displayed.

### ELISAs

To determine Vag8 binding to C1-inh, C1, C2, C3, C4 (all obtained from Complement Technology, Tyler, Texas, USA), alpha-1-antichymotrypsin (Biocentrum) and alpha-2-antiplasmin (Calbiochem), these proteins were coated on Immunolon plates over night at 4°C (all 3 μg/ml in PBS). All further steps were performed at 37°C, plates were washed 3 times with PBS with 0,05% T (PBS-T) between different steps. Plates were blocked by addition of PBS-T and 4% BSA (Serva) for 1 hour, followed by the addition of a three-fold concentration range of Vag8 starting at 30 μg/ml for 1 hour. Binding of Vag8 was detected by 1 μg/ml mouse-anti-6xhis (Hytest) followed by 0.2 μg/ml HRP-conjugated goat-anti-mouse secondary antibody (Southern Biotechnology Associates Inc). Complement ELISAs were performed as previously described [70] with minor modifications. For the CP and LP ELISA 1.25% NHS was used, whereas 40% NHS was used for the AP ELISA. The deposition of C5b-9 was detected using 1 μg/ml anti-C5b-9 (Abcam). Subsequently,0.2 μg/ml goat-anti-mouse-IgG-peroxidase(PO)-conjugated (Southern Biotechnology Associates Inc) was used for detection of anti-C4d and anti-C5b-9 while 0.125 μg/ml anti-DIG PO (Roche) was used for detection of anti-C3c. For both ELISA assays after final washes, HRP activity was detected by addition of TMB containing substrate solution, H_2_SO_4_ and subsequently read by an ELISA microplate reader (Bio-rad).

### Gel filtration chromatography

Gel filtration chromatography was performed to assess binding of Vag8 and C1-inh in fluid phase. 1 μM Vag8 and 1 μM C1-inh in PBS were run alone and together on the Superdex 200 increase 10/300 GL (GE Healthcare) connected to the ÄKTA-explorer (GE Healthcare).

### Complement activation on a bacterial surface

To determine the levels of complement deposition on a bacterial surface, heat inactivated *B*. *pertussis* B1917 (B1917) was used. Either Vag8 (60 μg/ml unless otherwise indicated) or Prn (60 μg/ml) (Sanofi), bacterial supernatant (50x concentrated) or OMVs (20 μg/ml) derived either from B1917 or B1917ΔVag8 was pre-incubated with 1.5% NHS in RPMI-HSA for 10 minutes after which 2x10^7^ bacteria were added and opsonisation was allowed to take place for 10 minutes (C2 detection) or 30 minutes (C4, C3 and C5b-9 detection) at 37°C 600 rpm. Subsequently, bacteria were spun down and supernatant was collected and mixed with equal volumes of 2X sample buffer(SB)containing 50 mM Dithiothreitol (SB-DTT) (Sigma) for complement protein detection by immunoblot as described below or bacteria were used for FACS analysis. For FACS analysis, the bacteria were washed with FACS buffer (PBS + 0.05% HSA) and complement deposition was determined using mouse-anti-human-C3b-FITC (3 μg/ml, Protos Immunoresearc), mouse-anti-human-C5b-9 (1 μg/ml, aE110, Santa Cruz) labelled with Alexa647 AlexaFluor 647 Antibody Labeling Kit, Molecular Probes by Life Technologies) or with mouse-anti-human-C4d (3 μg/ml, Quidel) followed by goat-anti-mouse-IgG-FITC (10 μg/ml, Dako, Santa Clara, CA, USA) at 4°C. Bacteria were fixed using 1.5% PFA, visualized using the FACSVerse (BD Biosciences) and analysed using FlowJo (Tree Star).

### Complement assay with purified components

C2 cleavage by C1s in the presence of C1-inh was analysed by incubating of C2 (50 μg/ml, Complement Technology), Vag8 (60 μg/ml), C1-inh (15 μg/ml Complement Technology) and C1s (0.15 μg/ml Complement Technology) for 30 minutes at 37°C. The reaction was stopped by addition of equal amounts of 2x SB and samples were boiled for 10 minutes. Samples were subjected to SDS-PAGE and visualized by Instant Blue (Gentaur) staining. To investigate the effect of Vag8 (20 μg/ml) on C1q (32 μg/ml, Complement Technology), C1s (6 μg/ml, Complement Technology), C1r (6,25 μg/ml, Complement Technology) and MASP-2 (6 μg/ml) binding to C1-inh (8 μg/ml, Complement Technology) in fluid phase, C1-inh was pre-incubated with or without Vag8 or Prn for 10 minutes at RT after which the protein of interest was added for 30 minutes at 37°C. The reaction was stopped by addition of 2x SB DTT and the effect was assessed by immunoblotting as described below by using anti-C1-inh (Sino Biologicals).

### Immunoblotting

All samples mixed with 2x SB or SB-DTT were boiled for 10 minutes, run on SDS-PAGE gels and transferred to PVDF membranes.Membranes were blocked with 4% skimmed milk in PBS-T and then incubated with a primary antibody either directed against C1-inh (0.3 μg/ml, rabbit-anti-human, Sino Biologicals, Beijing, China), C1r (1 μg/ml, goat-anti-human, R&D systems), C1s (2 μg/ml, goat-anti-human, Nordic Immunology), C1q (1 μg/ml, rabbit-anti-human, Dako), C4 (0.5 μg/ml, goat-anti-human, Complement Technology), C2 (3.3 μg/ml, Complement Technology) or C3 (2 μg/ml, goat-anti-human, Complement Technology). Subsequently the blots were incubated with the appropriate secondary antibody, goat-anti-rabbit-IgG-PO (0.5 μg/ml, Southern Biotechnology Associates Inc) or donkey-anti-goat-IgG-PO (0.5 μg/ml, Southern Biotechnology Associates Inc). All Antibodies were diluted in PBS-T-1% skimmed milk. For detection, the Pierce ECL Western Blotting Substrate (Thermofisher Scientific) was used and visualized using the ImageQuant (GE Life Sciences).

### Statistical analysis

Statistical analyses were performed using GraphPad Prism 6.02 and the differences between groups were analyzed for significance using the two-tailed Student's t-test. A p value of ≤0.05 was considered statistically significant.

## Supporting information

S1 Fig Correct refolding of recombinant Vag8. Circular dichroism spectrum of Vag8, showing a valley at 220 nm corresponding to a β-sheet which is as expected for correctly folded Vag8.
(TIF)

S2 Fig Gel filtration chromatography of Vag8 binding to C1-inh in fluid phase. (A) Chromatogram of Vag8 (light blue) and C1-inh (purple) separately and together (blue-purple) on a Superdex 200 Increase 10/300 GL column. The run of C1-inh and Vag8 together shows a higher molecular mass peak, suggesting complex formation of C1-inh and Vag8. Immunoblots of fractions B11, B12, C4 and C5 of A were analyzed with (B) anti-Vag8 or (C) anti-C1-inh. Fraction B11 contains both C1-inh and Vag8, showing complex formation. Fraction B12 contains predominantly C1-inh, corresponding to the C1-inh peak in panel A. C5 contains only Vag8, corresponding to the Vag8 peak in panel A. Figure is representative for three separate experiments.
(TIF)

S3 Fig C1s binding to C1 is inhibited by Vag8. C1r was detected using anti-C1r. The presence of C1r bound to C1 and C1-inh is inhibited in the presence of Vag8 but not Prn. Additionally, we show the presence of active C1r in the presence of Vag8. Figure is representative for three separate experiments.
(TIF)

S4 Fig Vag8 does not cleave C2. Purified C2 was incubated with Vag8 and visualized using Instant Blue. No cleavage is detected of C2 by Vag8. Figure is representative for three separate experiments.
(TIF)

S5 Fig C4 and C2 are degraded in the presence of Vag8. Incubation of 1.25% NHS alone with different concentrations of Vag8 shows cleavage of (A) C4 starting at 3.75 μg/ml Vag8 and (B) C2 starting at 7.5 μg/ml Vag8. Both figures are representative for three separate experiments.
(TIF)

## References

[1] Bart MJ, van Gent M, van der Heide HG, Boekhorst J, Hermans P, Parkhill J, et al Comparative genomics of prevaccination and modern Bordetella pertussis strains. BMC Genomics. 2010;11:627 doi: 10.1186/1471-2164-11-627 ; 21070624 10.1186/1471-2164-11-627 PMC3018138

[2] Lam C, Octavia S, Bahrame Z, Sintchenko V, Gilbert GL, Lan R. Selection and emergence of pertussis toxin promoter ptxP3 allele in the evolution of Bordetella pertussis. Infect Genet Evol. 2012;12(2):492–5. doi: 10.1016/j.meegid.2012.01.001 . 22293463 10.1016/j.meegid.2012.01.001

[3] Clarke M, McIntyre PB, Blyth CC, Wood N, Octavia S, Sintchenko V, et al The relationship between Bordetella pertussis genotype and clinical severity in Australian children with pertussis. J Infect. 2016;72(2):171–8. doi: 10.1016/j.jinf.2015.11.004 . 26675318 10.1016/j.jinf.2015.11.004

[4] Mooi FR, van Loo IH, van Gent M, He Q, Bart MJ, Heuvelman KJ, et al Bordetella pertussis strains with increased toxin production associated with pertussis resurgence. Emerg Infect Dis. 2009;15(8):1206–13. doi: 10.3201/eid1508.081511 ; 19751581 10.3201/eid1508.081511 PMC2815961

[5] Finn TM, Amsbaugh DF. Vag8, a Bordetella pertussis bvg-regulated protein. Infect Immun. 1998;66(8):3985–9. ; 9673293 10.1128/iai.66.8.3985-3989.1998 PMC108471

[6] Raeven RH, van der Maas L, Tilstra W, Uittenbogaard JP, Bindels TH, Kuipers B, et al Immunoproteomic Profiling of Bordetella pertussis Outer Membrane Vesicle Vaccine Reveals Broad and Balanced Humoral Immunogenicity. J Proteome Res. 2015;14(7):2929–42. doi: 10.1021/acs.jproteome.5b00258 . 25988566 10.1021/acs.jproteome.5b00258

[7] Marr N, Shah NR, Lee R, Kim EJ, Fernandez RC. Bordetella pertussis autotransporter Vag8 binds human C1 esterase inhibitor and confers serum resistance. PLoS One. 2011;6(6):e20585 doi: 10.1371/journal.pone.0020585 ; 21695123 10.1371/journal.pone.0020585 PMC3114845

[8] de Gouw D, Hermans PW, Bootsma HJ, Zomer A, Heuvelman K, Diavatopoulos DA, et al Differentially expressed genes in Bordetella pertussis strains belonging to a lineage which recently spread globally. PLoS One. 2014;9(1):e84523 doi: 10.1371/journal.pone.0084523 ; 24416242 10.1371/journal.pone.0084523 PMC3885589

[9] King AJ, van der Lee S, Mohangoo A, van Gent M, van der Ark A, van de Waterbeemd B. Genome-wide gene expression analysis of Bordetella pertussis isolates associated with a resurgence in pertussis: elucidation of factors involved in the increased fitness of epidemic strains. PLoS One. 2013;8(6):e66150 doi: 10.1371/journal.pone.0066150 ; 23776625 10.1371/journal.pone.0066150 PMC3679012

[10] Tan T, Dalby T, Forsyth K, Halperin SA, Heininger U, Hozbor D, et al Pertussis Across the Globe: Recent Epidemiologic Trends From 2000–2013. Pediatr Infect Dis J. 2015 doi: 10.1097/INF.0000000000000795 . 26376316 10.1097/INF.0000000000000795

[11] de Gouw D, de Jonge MI, Hermans PW, Wessels HJ, Zomer A, Berends A, et al Proteomics-identified Bvg-activated autotransporters protect against bordetella pertussis in a mouse model. PLoS One. 2014;9(8):e105011 doi: 10.1371/journal.pone.0105011 ; 25133400 10.1371/journal.pone.0105011 PMC4136822

[12] Jongerius I, Schuijt TJ, Mooi FR, Pinelli E. Complement evasion by Bordetella pertussis: implications for improving current vaccines. J Mol Med (Berl). 2015;93(4):395–402. doi: 10.1007/s00109-015-1259-1 ; 25686752 10.1007/s00109-015-1259-1 PMC4366546

[13] Persson CG, Erjefalt I, Alkner U, Baumgarten C, Greiff L, Gustafsson B, et al Plasma exudation as a first line respiratory mucosal defence. Clin Exp Allergy. 1991;21(1):17–24. . 2021873 10.1111/j.1365-2222.1991.tb00799.x

[14] Merle NS, Church SE, Fremeaux-Bacchi V, Roumenina LT. Complement System Part I—Molecular Mechanisms of Activation and Regulation. Front Immunol. 2015;6:262 doi: 10.3389/fimmu.2015.00262 ; 26082779 10.3389/fimmu.2015.00262 PMC4451739

[15] Gaboriaud C, Ling WL, Thielens NM, Bally I, Rossi V. Deciphering the fine details of c1 assembly and activation mechanisms: "mission impossible"? Front Immunol. 2014;5:565 doi: 10.3389/fimmu.2014.00565 ; 25414705 10.3389/fimmu.2014.00565 PMC4222235

[16] Ziccardi RJ. The first component of human complement (C1): activation and control. Springer Semin Immunopathol. 1983;6(2–3):213–30. . 6314572 10.1007/BF00205874

[17] Gal P, Ambrus G, Zavodszky P. C1s, the protease messenger of C1. Structure, function and physiological significance. Immunobiology. 2002;205(4–5):383–94. . 12396001 10.1078/0171-2985-00140

[18] Garred P, Genster N, Pilely K, Bayarri-Olmos R, Rosbjerg A, Ma YJ, et al A journey through the lectin pathway of complement-MBL and beyond. Immunol Rev. 2016;274(1):74–97. doi: 10.1111/imr.12468 . 27782323 10.1111/imr.12468

[19] Vorup-Jensen T, Jensenius JC, Thiel S. MASP-2, the C3 convertase generating protease of the MBLectin complement activating pathway. Immunobiology. 1998;199(2):348–57. doi: 10.1016/S0171-2985(98)80039-9 . 9777418 10.1016/S0171-2985(98)80039-9

[20] Thiel S, Vorup-Jensen T, Stover CM, Schwaeble W, Laursen SB, Poulsen K, et al A second serine protease associated with mannan-binding lectin that activates complement. Nature. 1997;386(6624):506–10. doi: 10.1038/386506a0 . 9087411 10.1038/386506a0

[21] Kjaer TR, Le le TM, Pedersen JS, Sander B, Golas MM, Jensenius JC, et al Structural insights into the initiating complex of the lectin pathway of complement activation. Structure. 2015;23(2):342–51. doi: 10.1016/j.str.2014.10.024 . 25579818 10.1016/j.str.2014.10.024

[22] Pangburn MK, Rawal N. Structure and function of complement C5 convertase enzymes. Biochem Soc Trans. 2002;30(Pt 6):1006–10. . 12440962 10.1042/bst0301006

[23] Janssen BJ, Christodoulidou A, McCarthy A, Lambris JD, Gros P. Structure of C3b reveals conformational changes that underlie complement activity. Nature. 2006;444(7116):213–6. doi: 10.1038/nature05172 . 17051160 10.1038/nature05172

[24] Morgan BP, Walters D, Serna M, Bubeck D. Terminal complexes of the complement system: new structural insights and their relevance to function. Immunol Rev. 2016;274(1):141–51. doi: 10.1111/imr.12461 . 27782334 10.1111/imr.12461

[25] Lambris JD, Ricklin D, Geisbrecht BV. Complement evasion by human pathogens. Nat Rev Microbiol. 2008;6(2):132–42. doi: 10.1038/nrmicro1824 ; 18197169 10.1038/nrmicro1824 PMC2814840

[26] Potempa M, Potempa J. Protease-dependent mechanisms of complement evasion by bacterial pathogens. Biol Chem. 2012;393(9):873–88. doi: 10.1515/hsz-2012-0174 ; 22944688 10.1515/hsz-2012-0174 PMC3488274

[27] Serruto D, Rappuoli R, Scarselli M, Gros P, van Strijp JA. Molecular mechanisms of complement evasion: learning from staphylococci and meningococci. Nat Rev Microbiol. 2010;8(6):393–9. doi: 10.1038/nrmicro2366 . 20467445 10.1038/nrmicro2366

[28] Hovingh ES, van den Broek B, Jongerius I. Hijacking Complement Regulatory Proteins for Bacterial Immune Evasion. Frontiers in Microbiology. 2016;7(2004). doi: 10.3389/fmicb.2016.02004 28066340 10.3389/fmicb.2016.02004 PMC5167704

[29] Wang M, Krauss JL, Domon H, Hosur KB, Liang S, Magotti P, et al Microbial hijacking of complement-toll-like receptor crosstalk. Sci Signal. 2010;3(109):ra11 doi: 10.1126/scisignal.2000697 ; 20159852 10.1126/scisignal.2000697 PMC2824906

[30] Zipfel PF, Skerka C. Complement regulators and inhibitory proteins. Nat Rev Immunol. 2009;9(10):729–40. doi: 10.1038/nri2620 . 19730437 10.1038/nri2620

[31] Marr N, Luu RA, Fernandez RC. Bordetella pertussis binds human C1 esterase inhibitor during the virulent phase, to evade complement-mediated killing. J Infect Dis. 2007;195(4):585–8. doi: 10.1086/510913 . 17230419 10.1086/510913

[32] Bart MJ, Zeddeman A, van der Heide HG, Heuvelman K, van Gent M, Mooi FR. Complete Genome Sequences of Bordetella pertussis Isolates B1917 and B1920, Representing Two Predominant Global Lineages. Genome Announc. 2014;2(6). doi: 10.1128/genomeA.01301-14 ; 25540342 10.1128/genomeA.01301-14 PMC4276820

[33] Everest P, Li J, Douce G, Charles I, De Azavedo J, Chatfield S, et al Role of the Bordetella pertussis P.69/pertactin protein and the P.69/pertactin RGD motif in the adherence to and invasion of mammalian cells. Microbiology. 1996;142 (Pt 11):3261–8. doi: 10.1099/13500872-142-11-3261 . 8969522 10.1099/13500872-142-11-3261

[34] Atosuo J, Lehtinen J, Vojtek L, Lilius EM. Escherichia coli K-12 (pEGFPluxABCDEamp): a tool for analysis of bacterial killing by antibacterial agents and human complement activities on a real-time basis. Luminescence. 2013;28(5):771–9. doi: 10.1002/bio.2435 . 23129448 10.1002/bio.2435

[35] Davis AE 3rd, Mejia P, Lu F. Biological activities of C1 inhibitor. Mol Immunol. 2008;45(16):4057–63. doi: 10.1016/j.molimm.2008.06.028 ; 18674818 10.1016/j.molimm.2008.06.028 PMC2626406

[36] Huntington JA, Read RJ, Carrell RW. Structure of a serpin-protease complex shows inhibition by deformation. Nature. 2000;407(6806):923–6. doi: 10.1038/35038119 . 11057674 10.1038/35038119

[37] Wijeyewickrema LC, Lameignere E, Hor L, Duncan RC, Shiba T, Travers RJ, et al Polyphosphate is a novel cofactor for regulation of complement by a serpin, C1 inhibitor. Blood. 2016;128(13):1766–76. doi: 10.1182/blood-2016-02-699561 ; 27338096 10.1182/blood-2016-02-699561 PMC5043130

[38] Locht C. Pertussis: acellular, whole-cell, new vaccines, what to choose? Expert Rev Vaccines. 2016;15(6):671–3. doi: 10.1586/14760584.2016.1161511 . 26938372 10.1586/14760584.2016.1161511

[39] Locht C, Papin JF, Lecher S, Debrie AS, Thalen M, Solovay K, et al Live attenuated pertussis vaccine BPZE1 protects baboons against B. pertussis disease and infection. J Infect Dis. 2017 doi: 10.1093/infdis/jix254 . 28535276 10.1093/infdis/jix254 PMC5853371

[40] Middendorf B, Stubs D, Guiso N, Deppisch H, Gross R, Fuchs TM. Phg, a novel member of the autotransporter family present in Bordetella species. Microbiol Res. 2005;160(3):329–36. doi: 10.1016/j.micres.2005.02.007 . 16035245 10.1016/j.micres.2005.02.007

[41] Junker M, Schuster CC, McDonnell AV, Sorg KA, Finn MC, Berger B, et al Pertactin beta-helix folding mechanism suggests common themes for the secretion and folding of autotransporter proteins. Proc Natl Acad Sci U S A. 2006;103(13):4918–23. doi: 10.1073/pnas.0507923103 ; 16549796 10.1073/pnas.0507923103 PMC1458770

[42] Grijpstra J, Arenas J, Rutten L, Tommassen J. Autotransporter secretion: varying on a theme. Res Microbiol. 2013;164(6):562–82. doi: 10.1016/j.resmic.2013.03.010 . 23567321 10.1016/j.resmic.2013.03.010

[43] Luu LD, Octavia S, Zhong L, Raftery M, Sintchenko V, Lan R. Characterisation of the Bordetella pertussis secretome under different media. J Proteomics. 2017;158:43–51. doi: 10.1016/j.jprot.2017.02.010 . 28242451 10.1016/j.jprot.2017.02.010

[44] Hozbor D, Rodriguez ME, Fernandez J, Lagares A, Guiso N, Yantorno O. Release of outer membrane vesicles from Bordetella pertussis. Curr Microbiol. 1999;38(5):273–8. . 10355115 10.1007/pl00006801

[45] Kang M, Ko YP, Liang X, Ross CL, Liu Q, Murray BE, et al Collagen-binding microbial surface components recognizing adhesive matrix molecule (MSCRAMM) of Gram-positive bacteria inhibit complement activation via the classical pathway. J Biol Chem. 2013;288(28):20520–31. doi: 10.1074/jbc.M113.454462 ; 23720782 10.1074/jbc.M113.454462 PMC3711317

[46] Garcia BL, Zwarthoff SA, Rooijakkers SH, Geisbrecht BV. Novel Evasion Mechanisms of the Classical Complement Pathway. J Immunol. 2016;197(6):2051–60. doi: 10.4049/jimmunol.1600863 ; 27591336 10.4049/jimmunol.1600863 PMC5012295

[47] Grosskinsky S, Schott M, Brenner C, Cutler SJ, Simon MM, Wallich R. Human complement regulators C4b-binding protein and C1 esterase inhibitor interact with a novel outer surface protein of Borrelia recurrentis. PLoS Negl Trop Dis. 2010;4(6):e698 doi: 10.1371/journal.pntd.0000698 ; 20532227 10.1371/journal.pntd.0000698 PMC2879370

[48] Barnes MG, Weiss AA. BrkA protein of Bordetella pertussis inhibits the classical pathway of complement after C1 deposition. Infect Immun. 2001;69(5):3067–72. doi: 10.1128/IAI.69.5.3067-3072.2001 ; 11292725 10.1128/IAI.69.5.3067-3072.2001 PMC98261

[49] Berggard K, Lindahl G, Dahlback B, Blom AM. Bordetella pertussis binds to human C4b-binding protein (C4BP) at a site similar to that used by the natural ligand C4b. Eur J Immunol. 2001;31(9):2771–80. . 11536176 10.1002/1521-4141(200109)31:9<2771::aid-immu2771>3.0.co;2-0

[50] Noofeli M, Bokhari H, Blackburn P, Roberts M, Coote JG, Parton R. BapC autotransporter protein is a virulence determinant of Bordetella pertussis. Microb Pathog. 2011;51(3):169–77. doi: 10.1016/j.micpath.2011.04.004 . 21554944 10.1016/j.micpath.2011.04.004

[51] Fernandez RC, Weiss AA. Serum resistance in bvg-regulated mutants of Bordetella pertussis. FEMS Microbiol Lett. 1998;163(1):57–63. . 9631546 10.1111/j.1574-6968.1998.tb13026.x

[52] Passerini de Rossi BN, Friedman LE, Gonzalez Flecha FL, Castello PR, Franco MA, Rossi JP. Identification of Bordetella pertussis virulence-associated outer membrane proteins. FEMS Microbiol Lett. 1999;172(1):9–13. . 10079522 10.1111/j.1574-6968.1999.tb13442.x

[53] Arnal L, Grunert T, Cattelan N, de Gouw D, Villalba MI, Serra DO, et al Bordetella pertussis Isolates from Argentinean Whooping Cough Patients Display Enhanced Biofilm Formation Capacity Compared to Tohama I Reference Strain. Front Microbiol. 2015;6:1352 doi: 10.3389/fmicb.2015.01352 ; 26696973 10.3389/fmicb.2015.01352 PMC4672677

[54] Bibova I, Hot D, Keidel K, Amman F, Slupek S, Cerny O, et al Transcriptional profiling of Bordetella pertussis reveals requirement of RNA chaperone Hfq for Type III secretion system functionality. RNA Biol. 2015;12(2):175–85. doi: 10.1080/15476286.2015.1017237 ; 25674816 10.1080/15476286.2015.1017237 PMC4615762

[55] Raeven RH, Brummelman J, van der Maas L, Tilstra W, Pennings JL, Han WG, et al Immunological Signatures after Bordetella pertussis Infection Demonstrate Importance of Pulmonary Innate Immune Cells. PLoS One. 2016;11(10):e0164027 doi: 10.1371/journal.pone.0164027 . 27711188 10.1371/journal.pone.0164027 PMC5053408

[56] Otsuka N, Gotoh K, Nishimura N, Ozaki T, Nakamura Y, Haga K, et al A Novel IgM-capture enzyme-linked immunosorbent assay using recombinant Vag8 fusion protein for the accurate and early diagnosis of Bordetella pertussis infection. Microbiol Immunol. 2016;60(5):326–33. doi: 10.1111/1348-0421.12378 . 26996337 10.1111/1348-0421.12378

[57] Kaplan AP, Joseph K. The bradykinin-forming cascade and its role in hereditary angioedema. Ann Allergy Asthma Immunol. 2010;104(3):193–204. doi: 10.1016/j.anai.2010.01.007 . 20377108 10.1016/j.anai.2010.01.007

[58] Burman JD, Leung E, Atkins KL, O'Seaghdha MN, Lango L, Bernado P, et al Interaction of human complement with Sbi, a staphylococcal immunoglobulin-binding protein: indications of a novel mechanism of complement evasion by Staphylococcus aureus. J Biol Chem. 2008;283(25):17579–93. doi: 10.1074/jbc.M800265200 ; 18434316 10.1074/jbc.M800265200 PMC2649420

[59] Laarman AJ, Ruyken M, Malone CL, van Strijp JA, Horswill AR, Rooijakkers SH. Staphylococcus aureus metalloprotease aureolysin cleaves complement C3 to mediate immune evasion. J Immunol. 2011;186(11):6445–53. doi: 10.4049/jimmunol.1002948 . 21502375 10.4049/jimmunol.1002948

[60] Barnes MG, Weiss AA. Activation of the complement cascade by Bordetella pertussis. FEMS Microbiol Lett. 2003;220(2):271–5. . 12670691 10.1016/S0378-1097(03)00132-0

[61] Thalen M, van den IJ, Jiskoot W, Zomer B, Roholl P, de Gooijer C, et al Rational medium design for Bordetella pertussis: basic metabolism. J Biotechnol. 1999;75(2–3):147–59. . 10553654 10.1016/s0168-1656(99)00155-8

[62] Stibitz S, Yang MS. Subcellular localization and immunological detection of proteins encoded by the vir locus of Bordetella pertussis. J Bacteriol. 1991;173(14):4288–96. ; 2066330 10.1128/jb.173.14.4288-4296.1991 PMC208088

[63] Stibitz S, Black W, Falkow S. The construction of a cloning vector designed for gene replacement in Bordetella pertussis. Gene. 1986;50(1–3):133–40. . 2884169 10.1016/0378-1119(86)90318-5

[64] Choi KH, Gaynor JB, White KG, Lopez C, Bosio CM, Karkhoff-Schweizer RR, et al A Tn7-based broad-range bacterial cloning and expression system. Nat Methods. 2005;2(6):443–8. doi: 10.1038/nmeth765 . 15908923 10.1038/nmeth765

[65] Bardoel BW, Vos R, Bouman T, Aerts PC, Bestebroer J, Huizinga EG, et al Evasion of Toll-like receptor 2 activation by staphylococcal superantigen-like protein 3. J Mol Med (Berl). 2012;90(10):1109–20. doi: 10.1007/s00109-012-0926-8 . 22714643 10.1007/s00109-012-0926-8

[66] Oliver DC, Huang G, Nodel E, Pleasance S, Fernandez RC. A conserved region within the Bordetella pertussis autotransporter BrkA is necessary for folding of its passenger domain. Mol Microbiol. 2003;47(5):1367–83. . 12603741 10.1046/j.1365-2958.2003.03377.x

[67] van Gent M, van Loo IH, Heuvelman KJ, de Neeling AJ, Teunis P, Mooi FR. Studies on Prn variation in the mouse model and comparison with epidemiological data. PLoS One. 2011;6(3):e18014 doi: 10.1371/journal.pone.0018014 ; 21464955 10.1371/journal.pone.0018014 PMC3064647

[68] Saunders NB, Shoemaker DR, Brandt BL, Moran EE, Larsen T, Zollinger WD. Immunogenicity of intranasally administered meningococcal native outer membrane vesicles in mice. Infect Immun. 1999;67(1):113–9. ; 9864204 10.1128/iai.67.1.113-119.1999 PMC96285

[69] Zollinger WD, Donets MA, Schmiel DH, Pinto VB, Labrie JE, Moran EE, et al Design and evaluation in mice of a broadly protective meningococcal group B native outer membrane vesicle vaccine. Vaccine. 2010;28(31):5057–67. doi: 10.1016/j.vaccine.2010.05.006 20653107 10.1016/j.vaccine.2010.05.006

[70] Seelen MA, Roos A, Wieslander J, Mollnes TE, Sjoholm AG, Wurzner R, et al Functional analysis of the classical, alternative, and MBL pathways of the complement system: standardization and validation of a simple ELISA. J Immunol Methods. 2005;296(1–2):187–98. doi: 10.1016/j.jim.2004.11.016 . 15680163 10.1016/j.jim.2004.11.016

