# Supplementary figures and images for: Acquisition of C1 inhibitor by Bordetella pertussis virulence associated gene 8 results in C2 and C4 consumption away from the bacterial surface
Source: PLoS Pathog. 2017 Jul 24;13(7):e1006531. doi: 10.1371/journal.ppat.1006531 (PMC5542704; doi:10.1371/journal.ppat.1006531)

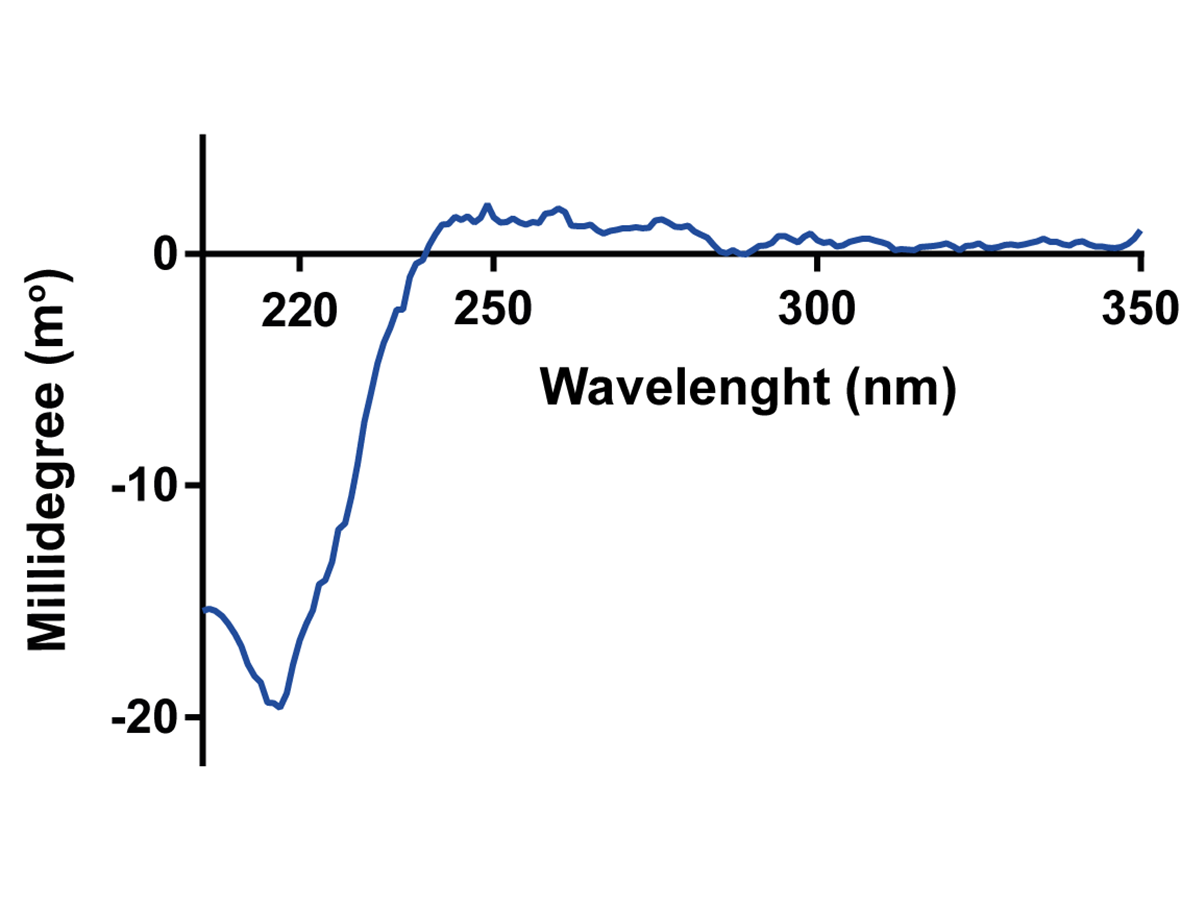

Supplement: S1 Fig — Circular dichroism spectrum of Vag8, showing a valley at 220 nm corresponding to a β-sheet which is as expected for correctly folded Vag8. (TIF) [file ppat.1006531.s001.tif]

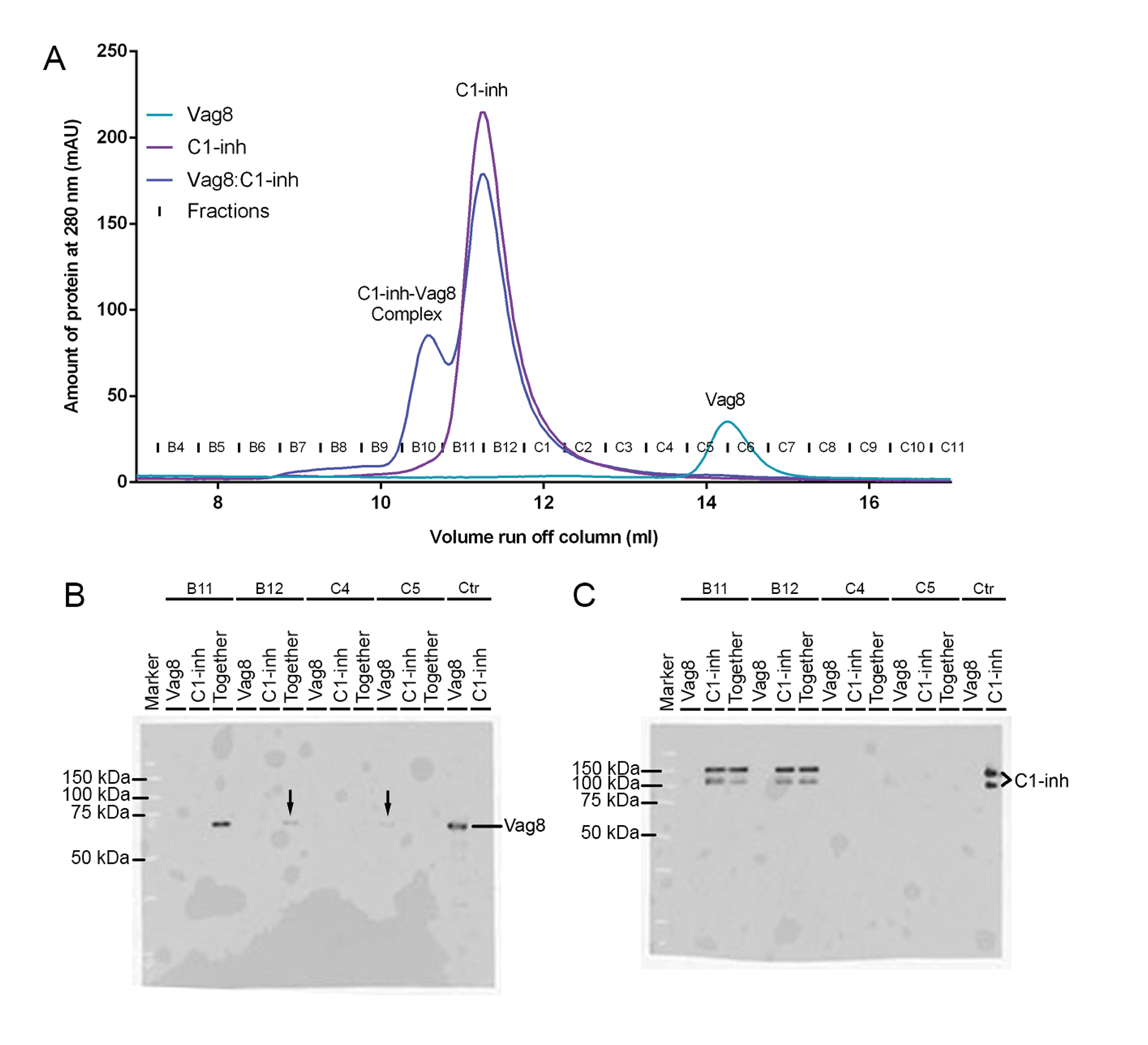

Supplement: S2 Fig — (A) Chromatogram of Vag8 (light blue) and C1-inh (purple) separately and together (blue-purple) on a Superdex 200 Increase 10/300 GL column. The run of C1-inh and Vag8 together shows a higher molecular mass peak, suggesting complex formation of C1-inh and Vag8. Immunoblots of fractions B11, B12, C4 and C5 of A were analyzed with (B) anti-Vag8 or (C) anti-C1-inh. Fraction B11 contains both C1-inh and Vag8, showing complex formation. Fraction B12 contains predominantly C1-inh, corresponding to the C1-inh peak in panel A. C5 contains only Vag8, corresponding to the Vag8 peak in panel A. Figure is representative for three separate experiments. (TIF) [file ppat.1006531.s002.tif]

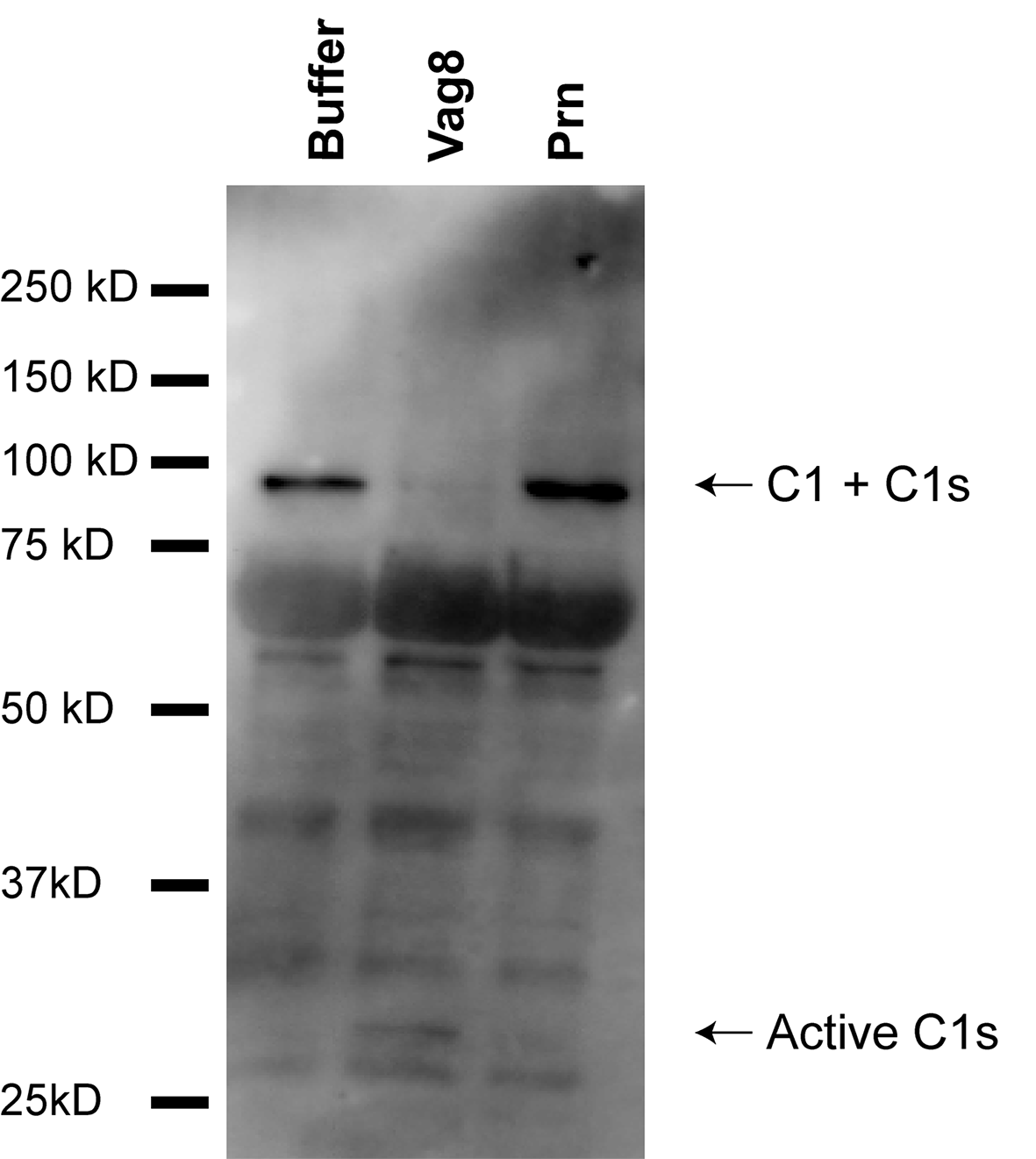

Supplement: S3 Fig — C1r was detected using anti-C1r. The presence of C1r bound to C1 and C1-inh is inhibited in the presence of Vag8 but not Prn. Additionally, we show the presence of active C1r in the presence of Vag8. Figure is representative for three separate experiments. (TIF) [file ppat.1006531.s003.tif]

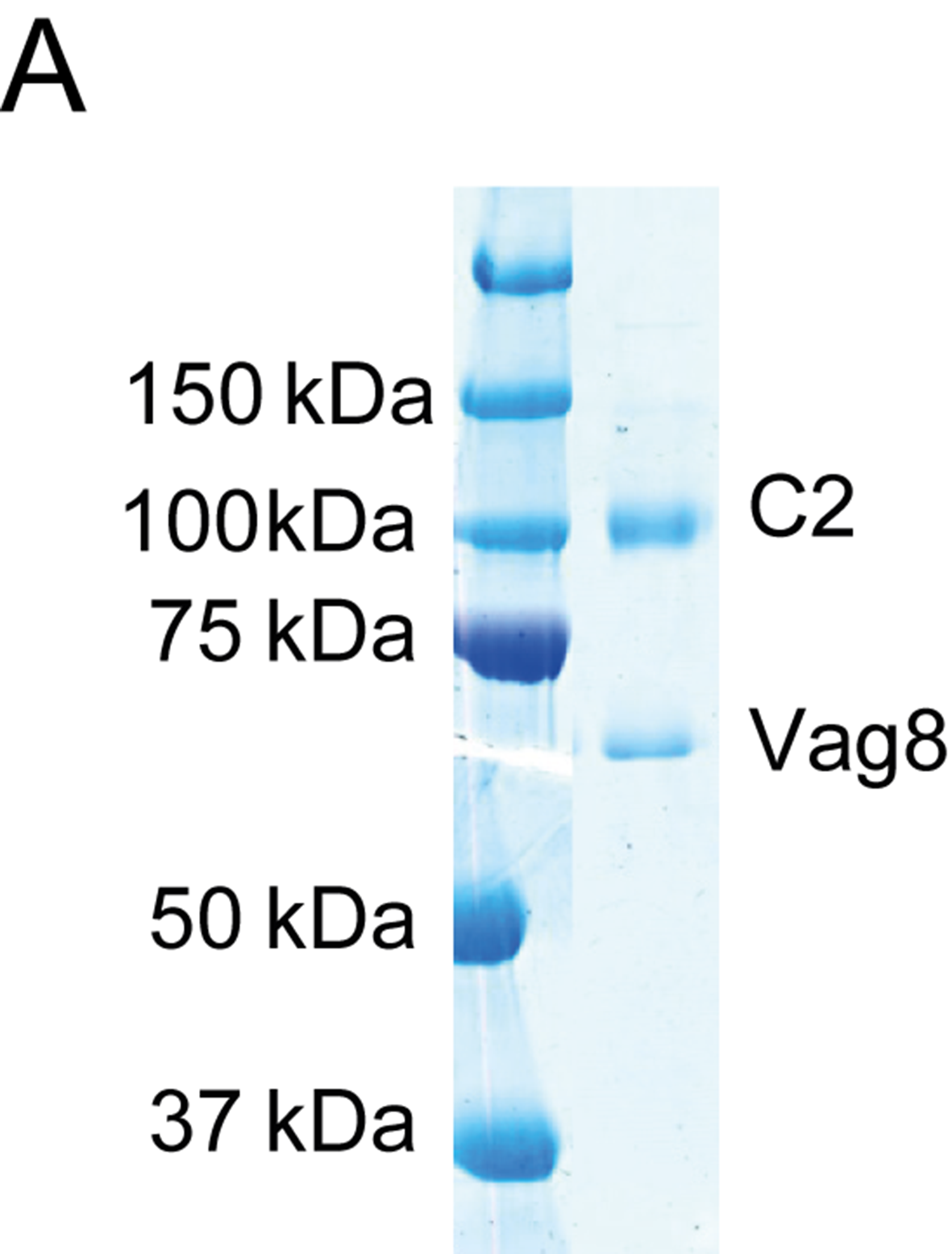

Supplement: S4 Fig — Purified C2 was incubated with Vag8 and visualized using Instant Blue. No cleavage is detected of C2 by Vag8. Figure is representative for three separate experiments. (TIF) [file ppat.1006531.s004.tif]

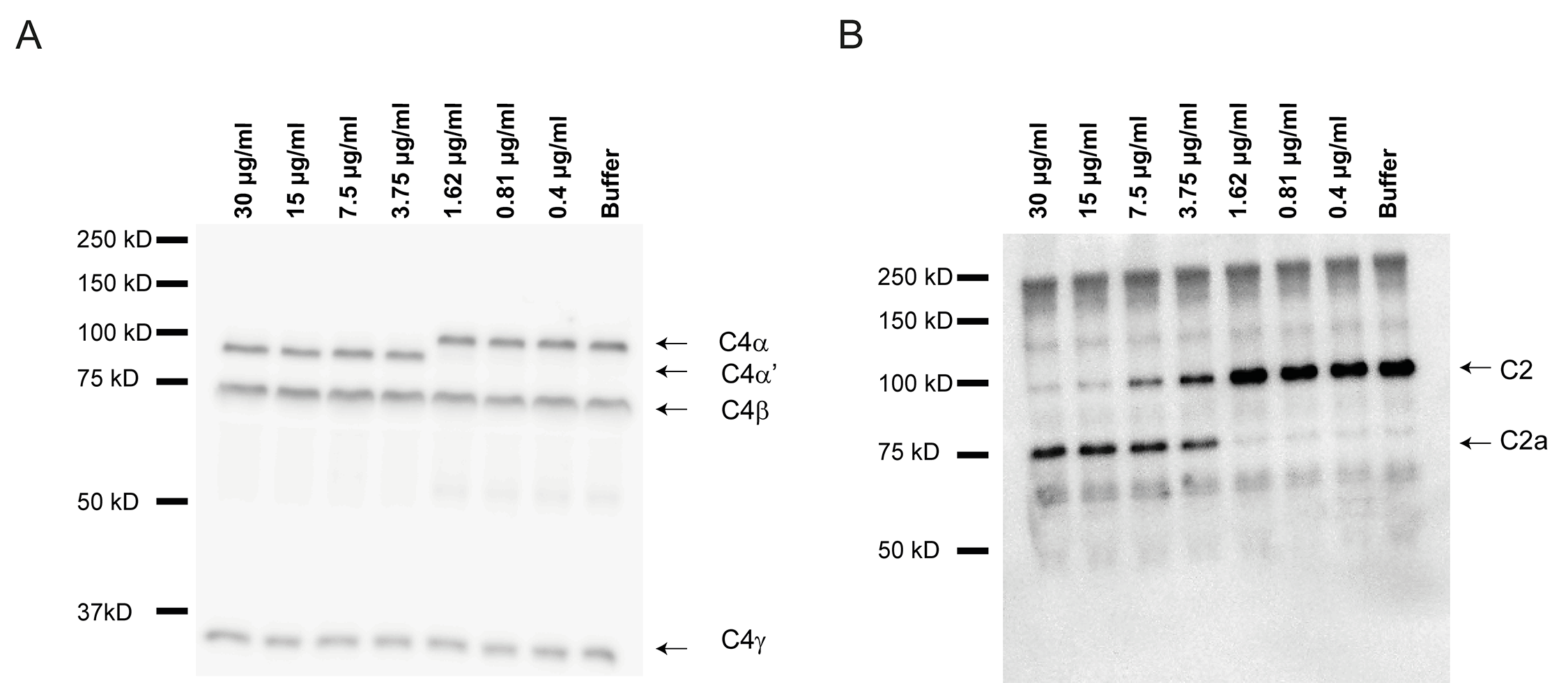

Supplement: S5 Fig — Incubation of 1.25% NHS alone with different concentrations of Vag8 shows cleavage of (A) C4 starting at 3.75 μg/ml Vag8 and (B) C2 starting at 7.5 μg/ml Vag8. Both figures are representative for three separate experiments. (TIF) [file ppat.1006531.s005.tif]
